# Regioselective Synthesis, Characterization, and Antimicrobial Activities of Some New Monosaccharide Derivatives

**DOI:** 10.3797/scipharm.1308-03

**Published:** 2013-09-26

**Authors:** Sarkar M. A. Kawsar, Md O. Faruk, Mohammad S. Rahman, Yuki Fujii, Yasuhiro Ozeki

**Affiliations:** 1 Laboratory of Carbohydrate and Protein Chemistry, Department of Chemistry, Faculty of Science, University of Chittagong, Chittagong-4331, Bangladesh.; 2 Department of Microbiology, Faculty of Biological Science, University of Chittagong, Chittagong-4331, Bangladesh.; 3 Divisions of Microbiology and Functional Morphology, Department of Pharmacy, Faculty of Pharmaceutical Science, Nagasaki International University, 2825-7 Huis Ten Bosch, Sasebo, Nagasaki 859-3298, Japan.; 4 Laboratory of Glycobiology and Marine Biochemistry, Department of Genome System Sciences, Graduate School of Nanobiosciences, Yokohama City University, Yokohama 236-0027, Japan.

**Keywords:** Glucopyranosides, Synthesis, Derivatives, Elucidation, Spectroscopy, Antimicrobial

## Abstract

A regioselective acylation series of methyl α-D-glucopyranoside (**1**), methyl 3-*O*-benzoyl-4,6-*O*-benzylidene-α-D-mannopyranoside (**1A**), and methyl 4,6-*O*-benzylidene-2-*O*-(3,5-dinitrobenzoyl)-α-D-mannopyranoside (**1B**) has been carried out by the direct acylation method and afforded the 2,6-di-*O*-glucopyranoside and 2 or 3-*O*-mannopyranoside derivatives in an excellent yield. In order to obtain newer products, the 2,6-di-*O*-glucopyranoside derivative was further transformed to a series of 3,4-di-*O*-acyl derivatives containing a wide variety of functionalities in a single molecular framework. The structures of the newly synthesized compounds were elucidated on the basis of IR, ^1^H-NMR, ^13^C-NMR, ^13^C-DEPT spectral data, and elemental analysis. These synthesized derivatives were screened for *in vitro* antimicrobial activities against ten human pathogenic and five phytopathogenic microorganisms. A number of test compounds showed remarkable antimicrobial activity comparable to, and in some cases even higher than, the standard antibiotics employed. It was observed that methyl 3,4-di-*O*-(3-chlorobenzoyl)-2,6-di-*O*-hexanoyl-α-D-glucopyranoside (**8**) exhibited a varied range of MIC from 12.5 μg/disc to 25 μg/disc by the disk diffusion method and 1000 μg/mL to 1250 μg/mL by the broth macrodilution method.

## Introduction

Carbohydrate chemistry is now found in the field of organic synthesis, protein and nucleic acid chemistry, enzymology, antibiotics, immunology, and biotechnology. Carbohydrates also play an important role in our industrial development and many industries are based on the utilization of carbohydrates. With the development of modern and sophisticated techniques, the isolation of various natural products from plants and other sources become easier. Of the carbohydrates isolated from natural sources, acyl- and alkyl-glycoses or glycoside derivatives are important and some of these have effective biological activity [1–3]. Selective acylation is considered by carbohydrate chemists as one of the most useful and versatile methods for the preparation of the hydroxyl groups [4, 5]. Various selective acylation methods have so been developed and employed successfully in carbohydrate chemistry [6, 7]. Of these, the direct method has been considered by the carbohydrate chemists as one of the most effective for selective acylation of carbohydrates. Selective acylation is also important because of its usefulness for the synthesis of biologically active carbohydrates [8–10] and nucleosides [11, 12]. From the literature survey, it was revealed that a large number of biologically active compounds possess aromatic and heteroaromatic nuclei [13–15]. The benzene and substituted benzene nuclei play an important role as the common denominator for various biological activities [16, 17]. Nitrogen (N)- and sulphur (S)-containing substitution products also showed marked antimicrobial activities [18, 19]. As a continuation of a research project on the biological evaluation of carbohydrate derivatives and guided by some encouraging results obtained in this field [20–22], we deliberately synthesized some acylated derivatives of D-glucopyranoside (Scheme 1) and D-mannopyranoside (Scheme 2) containing a variety of substituents in a single molecular framework. We also evaluated the antibacterial and antifungal activities of the synthesized compounds using various bacterial and fungal strains and the results are reported here for the first time.

## Results and Discussion

### Synthesis

The main aim of the present work reported here was to study selective acylation of D-glucopyranoside (Scheme 1) and D-mannopyranoside (Scheme 2) by the direct acylation method using some non-traditional acylating agents, namely hexanoyl chloride, methanesulphonyl chloride, 4-methoxybenzoyl chloride, 4-nitrobenzoyl chloride, 4-chlorobenzoyl chloride, 3-chlorobenzoyl chloride, and pentanoyl chloride. The structure of the various suitably substituted monosaccharide derivatives were ascertained by analyzing their IR, ^1^H-NMR, and ^13^C-NMR spectra. All the acylation products were employed as test chemicals for antibacterial and antifungal screening studies against a number of human pathogenic bacteria and plant pathogenic fungi. Our initial effort was to carry out regioselective hexanoylation of methyl α-D-glucopyranoside (**1**). A number of derivatives of the resulting hexanoylation product were also prepared in order to achieve supportive evidences for structure elucidation and also to obtain newer derivatives of synthetic and biological importance.

### Spectral Characterization

Thus, the treatment of methyl α-D-glucopyranoside (**1**) with 1.1 molar equivalent of hexanoyl chloride in pyridine under freezing conditions, followed by the usual work-up and separation by silica gel column chromatography, afforded compound **2** in 45% yield as a syrup. Its IR spectrum showed absorption bands at 1730 cm^−1^ (-CO stretching) and 3610 cm^−1^ (-OH group). In its ^1^H-NMR spectrum, the C-2 proton resonated at δ 4.64 (as dd, J = 3.7 and 10.0 Hz) and shifted downfield from its original value (~ 4.00 ppm). The C-6 protons also shifted downfield to δ 4.38 (as dd, J = 5.0 and 12.1 Hz, 6a) and δ 4.27 (as dd, J = 2.0 and 12.1 Hz, H-6b) as compared to the starting glucopyranoside (**2**) (~ 3.5 ppm). The rest of the protons resonated in their anticipated positions, thereby suggesting that the hexanoyl group was introduced at positions 2 and 6. In its ^1^H-NMR spectrum, the presence of two two-proton triplets at δ 2.35 & 2.33, a four-proton multiplet at δ 1.60, an eight-proton multiplet at δ 1.27, and a six-proton triplet at δ 0.85 were indicative of the introduction of two hexanoyl groups in the molecule. The presence of two hexanoyl groups in the molecule was also shown by its ^13^C-NMR spectrum which displayed the following characteristic peaks: δ 174.51, δ 173.76 {2×CH_3_(CH_2_)_4_*C*O-}, δ 34.09, 34.05, 31.21, 31.12, 24.53, 24.44, 22.27, 22.22 {2×CH_3_(*C*H_2_)_4_CO-}, and δ 13.83, 13.81 {2×*C*H_3_(CH_2_)_4_CO-}. From the ^13^C-DEPT spectrum, it was found that there are nine methylene carbons which corresponded to 2× CH_3_(*C*H_2_)_4_CO- and C-6 of the glucose molecule. Complete analysis of the IR, ^1^H- and ^13^C-NMR spectra enabled us to ascertain its structure as methyl 2,6-di-*O*-hexanoyl-α-D-glucopyranoside (**2**). The structure of the hexanoyl derivative (**2**) was further ascertained by its conversion to and identification of its acetyl derivative (**3**). Thus, the reaction of compound **2** with an excess of acetic anhydride in pyridine, followed by the usual work-up procedure and silica gel column chromatographic purification, provided the acetyl derivative (**3**) in 92% yield as a syrup. The IR spectrum displayed the characteristic absorption band at 1710 cm^−1^ (for –CO stretching) in the molecule. The introduction of two acetyl groups in the molecule was demonstrated by the appearance of two three-proton singlets at δ 1.99 and 1.96 in its ^1^H-NMR spectrum. The C-3 proton resonated at δ 5.45 (as t, J = 9.8 Hz) and shifted downfield from the precursor diol (**2**) (δ 3.90); also, the C-4 proton resonated downfield to δ 5.02 (as t, J = 9.9 Hz) as compared to the precursor compound **2** (δ 3.72), thereby suggesting the attachment of the acetyl groups at positions 3 and 4. In its ^13^C-NMR spectra, the presence of the peaks at δ 169.95, δ 169.51 (2× CH_3_*C*O-), and δ 20.63, δ 20.58 (2× *C*H_3_CO-), also supported the presence of two acetyl groups in the molecule. By complete analysis of the IR, ^1^H-NMR, ^13^C-NMR, and ^13^C-DEPT spectra, the structure of the diacetate was ascertained as methyl 3,4-di-*O*-acetyl-2,6-di-*O-*hexanoyl-α-D-glucopyranoside (**3**). The structure of compound **2** was also supported by its transformation to and identification of the methanesulphonyl derivative (**4**). Compound **4** was prepared in 95% yield as a syrup by using methanesulphonyl chloride in pyridine at freezing temperature. Its IR spectrum showed an absorption band at 1720 cm^−1^ for –CO stretching. The presence of two methanesulphonyl groups in the molecule was demonstrated by its ^1^H-NMR spectrum which displayed two three-proton singlets at δ 3.14 and δ 3.10 due to the methyl protons of two methanesulphonyloxy groups. Also, the C-3 and C-4 protons shifted downfield to δ 5.10 (as t, J = 9.4 Hz) and δ 4.80 (as t, J = 9.4 Hz) from its precursor compound **2** (δ 3.90 and δ 3.72), thereby suggesting the attachment of the methanesulphonyl groups at positions 3 and 4. Its ^13^C-NMR spectrum displayed the characteristic peaks at δ 39.08 and 38.97 (2× *C*H_3_SO_2_-) due to the presence of two methanesulphonyl groups. By analysis of the IR, ^1^H-NMR, ^13^C-NMR, and ^13^C-DEPT spectra, that led us to establish its structure as methyl 2,6-di-*O*-hexanoyl-3,4-di-*O*-methanesulphonyl-*α*-D-glucopyranoside (**4**). We then performed 4-methoxybenzoylation of compound **2** using similar procedures and isolated compound **5** in 81% yield as a semi-solid mass. The IR spectrum of this compound **5** displayed an absorption band at 1690 cm^−1^ due to the carbonyl stretching. In its ^1^H-NMR spectrum, four characteristic doublets at δ 8.07, 7.84, 6.96, 6.80, and a six-proton singlet at δ 3.87 indicated the presence of two 4-methoxybenzoyl groups in the molecule. The attachment of the 4-methoxybenzoyl groups at C-3 and C-4 was confirmed by observing considerable downfield shifts of H-3 to δ 5.89 (as t, J = 9.8 Hz) and H-4 to δ 5.42 (as t, J = 9.6 Hz) in its ^1^H-NMR spectrum. Its ^13^C-NMR spectrum displayed *inter alia* the following characteristic peaks: δ 164.90, δ 162.26 (2× 4-OCH_3_.C_6_H_4_*C*O-), δ 132.79 (×4), 131.92, 131.80, 114.13 (×4), 113.65, 113.56 (2× 4-OCH_3_.*C*_6_H_4_*C*O-). The hexanoyl derivative (**2**) was then converted to the di-*O*-(4-nitrobenzoyl), di-*O*-(4-chlorobenzoyl), and di-*O*-(3-chlorobenzoyl) derivatives (**6**–**8**) by using a similar reaction and work-up procedures. The structures of these derivatives (**6**–**8**) were confidently assigned by completely analyzing their IR, ^1^H- NMR, ^13^C-NMR, and ^13^C-DEPT spectra. In all the cases, the introduction of the substituents at positions 3 and 4 were ascertained. We then employed pentanoyl chloride for derivatizing compound **2**. By using the conventional reaction, work-up, and purification procedure, we isolated the pentanoyl derivative (**9**) in 86% yield as thick syrup. By complete analysis of its IR, ^1^H-NMR, ^13^C-NMR, and ^13^C-DEPT spectra, the structure of the pentanoyl derivative was elucidated as methyl 2,6-di-*O*-hexanoyl-3,4-di-*O*-pentanoyl-α-D-glucopyranoside (**9**).

We then used a number of partially substituted monosaccharide derivatives, synthesized earlier in this laboratory, for acylation with a number of acylating agents in order to attain newer test chemicals for antimicrobial evaluation studies. Thus, pentanoylation of methyl 3-*O*-benzoyl-4,6-*O*-benzylidene-α-D-mannopyranoside (**1A**) with pentanoyl chloride in pyridine gave compound **10** in 90% yield which is very similar to [23]. The IR spectrum of this compound indicated an absorption band at 1718 cm^−1^ corresponding to carbonyl stretching. In its ^1^H-NMR spectrum, three two-proton multiplets at δ 2.41, 1.59, 1.32, and a three-proton multiplet at δ 0.86 corresponded to the presence of one pentanoyl group in the molecule. The introduction of the pentanoyl group at position 2 was demonstrated by a downfield shift of H-2 to δ 5.48. The ^13^C-NMR spectrum of compound **10** also showed the presence of one pentanoyl group by displaying the following characteristic peaks: δ 172.52 {CH_3_(CH_2_)_3_*C*O*-*}, δ 33.85, 26.98, 22.11 {CH_3_(*C*H_2_)_3_CO*-*}, and δ 13.62 {*C*H_3_(CH_2_)_3_CO*-*}. The benzylidene derivative (**1A**) was then derivatized by using hexanoyl chloride in pyridine followed by the usual work-up and purification procedures. The hexanoyl derivative (**11**) was isolated in 92% yield as syrup. The ^1^H-NMR spectrum of this compound showed the following characteristic peaks: δ 2.40 (2H, t, J = 7.4 Hz), δ 1.60 (2H, m), δ 1.27 {4H, m), and δ 0.86 {3H, m) ascertaining the presence of one hexanoyl group. The downfield shift of H-2 to δ 5.48 indicated the attachment of the hexanoyl group at position 2. In its ^13^C-NMR spectrum, the resonance peaks at δ 172.59 {CH_3_(CH_2_)_4_*C*O*-*}, δ 34.15, 31.18, 24.62, 22.27 {CH_3_(*C*H_2_)_4_CO*-*}, and δ13.84 {*C*H_3_(CH_2_)_4_CO*-*} were due to the presence of one hexanoyl group in the molecule.

The next substituted monosaccharide we used was methyl 4,6-*O*-benzylidene-2-*O*-(3,5-dinitrobenzoyl)-α-D-mannopyranoside (**1B**) and employed pentanoyl chloride, hexanoyl chloride, and 4-methoxybenzoyl chloride as the acylating agents. Thus, the benzylidene derivative (**1B**) upon treatment with pentanoyl chloride in pyridine, followed by the usual work-up and purification, afforded the pentanoyl derivative (**12**) in 91% yield as needles, m.p. 142–143°C. The IR spectrum of this compound displayed absorption band at 1711 cm^−1^ which corresponded to a carbonyl group. In its ^1^H- NMR spectrum, the appearance of the resonance peaks at δ 2.27 (2H, m), δ 1.47 (2H, t, J= 7.5 Hz), δ 1.16 (2H, m), and δ 0.69 (3H, t, J= 7.3 Hz) and the downfield shift of H-3 to δ 5.79 (as t, J = 9.5 Hz) indicated the attachment of one pentanoyl group at position 3. In its ^13^C-NMR spectrum, the appearance of resonance peaks at δ 172.75 {CH_3_(CH_2_)_3_*C*O*-*}, δ 33.95, 27.03, 22.02 {CH_3_(*C*H_2_)_3_CO*-*}, and δ 13.52 {*C*H_3_(CH_2_)_3_CO*-*} corresponded to one pentanoyl group.

Hexanoylation of the benzylidene derivative (**1B**) with hexanoyl chloride in pyridine provided compound **13** in 82% yield as needles, m.p. 137–138°C. The IR spectrum of this compound showed the following characteristic peak: 1712 cm^−1^ for –CO stretching. The introduction of one hexanoyl group was established by observing the following characteristic peaks in its ^1^H- NMR spectrum: δ 2.26 (2H, m), δ 1.49 (2H, t, J= 7.1 Hz), δ 1.11 (4H, m), and δ 0.67 (3H, t, J= 6.7 Hz). Also, we observed the downfield shift of H-3 to δ 5.79 (as t, J = 9.4 Hz) showing the introduction of the hexanoyl group at position 3. Its ^13^C-NMR spectrum also showed the presence of one hexanoyl group by displaying the following characteristic peaks: δ 172.80 {CH_3_(CH_2_)_4_*C*O*-*}, δ 34.19, 31.02, 24.66, 22.14 {CH_3_(*C*H_2_)_4_CO*-*}, and δ 13.68 {*C*H_3_(CH_2_)_4_CO*-*}. The benzylidene derivative (**1B**) was then allowed to react with 4-methoxybenzoyl chloride in pyridine and after the usual work-up and chromatographic purification, we obtained compound **14** in 85% yield as syrup. The IR spectrum of compound **14** showed an absorption band at 1716 cm^−1^ (-CO stretching), thereby suggesting the presence of a carbonyl group in the molecule. In its ^1^H-NMR spectrum, the characteristic two-proton doublets at δ 8.08 and δ 6.97 (J = 8.8 Hz in each case) and a three-proton singlet at δ 3.88 were due to one 4-methoxybenzoyl group in the molecule. Also, the deshielding of H-3 to δ 6.04 (as t, J = 9.5 Hz) indicated the formation of the 3-substitution product. The ^13^C-NMR spectrum also displayed the introduction of one 4-methoxybenzoyl group. Thus, the regioselective acylation of **D**-glucopyranoside (Scheme-1) and **D**-mannopyranoside (Scheme-2) by applying the direct acylation method was unique in that the reaction provided a single monosubstitution product in reasonably high yields.

### Anibacterial Activity

**Fig. 1:**
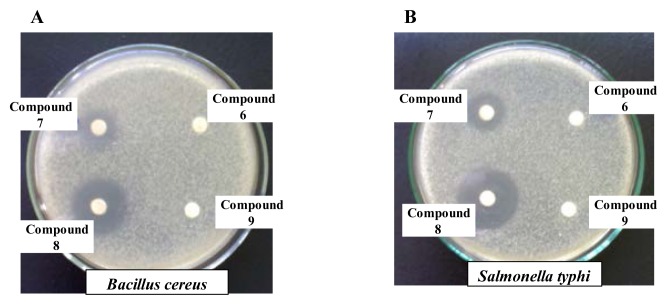
Zone of inhibition against *B. cereus* (A) *and S. typhi* (B) by the compounds **6**–**9**.

The antibacterial evaluation results of the test compounds and the standard antibiotic, ampicillin, against Gram-positive and Gram-negative bacteria are listed in Table 1, Table 2, and Fig 1, respectively.

From the results, we observed that **2**, **5**, **7**, **8,** and **14** were very sensitive towards all of both Gram-positive and Gram-negative bacterial organisms. Of the acylated derivatives, compound **8** was found to have the highest antibacterial functionality against all tested microorganisms viz., *B. subtilis* (20 mm), *B. cereus* (26 mm), *B. megaterium* (28 mm), *S. aureus* (27 mm), *P.* species (21 mm), *S. typhi* (28 mm), *S. paratyphi* (25 mm), *S. sysenteriae* (25 mm), *S. sonnei* (22 mm), and INABAET (*Vibrio*) (28 mm) which was much more than that of the standard antibiotic, ampicillin. In the case of **2**, *B. megaterium* (20 mm), *S. Typhi* (20 mm), **5**, *B. megaterium* (12 mm), **7**, *S. Typhi* (16 mm), and **14**, *B. megaterium* (20 mm), were found to be very sensitive. So, these compounds may be targeted in future studies for their usage as broad-spectrum antibiotics. Most of the test compounds showed mild inhibition and some were unable to show any inhibition at all against the tested microorganisms. In general, it has been observed that the average antibacterial results of the compounds for Gram-positive microorganisms follows the pattern: **8** > **7** > **2** > **14** > **5**, whereas Gram-negative bacteria follow the order: **8** > **7** > **2** > **14** > **5**.

From the minimum inhibition concentration (MIC) experimental results, it was observed that chemical **8** exhibited **a** varied range of values from 12.5 μg/disc to 25 μg/disc and 1000 μg/mL to 1250 μg/mL by the disk diffusion (Table 3) and broth macrodilution methods, (Table 4) respectively. The lowest MIC (12.5 μg/disc) was recorded against *B. cereus*, *B. megaterium*, *S. aureus*, *S. Typhi,* and INABAET (*Vibrio*) by the disk diffusion method and the lowest MIC (1000 μg/mL) was recorded against *B. cereus*, *B. megaterium*, *S. Aureus,* and INABAET (*Vibrio*) by the broth macrodilution method. The MIC is indicative of the usefulness of these compounds as antimicrobial drugs, but some other experiments must be carried out before these can be used as effective drugs. As chemical **8** exhibited remarkable inhibitory activity against ten pathogenic bacteria, the efficacy of the chemical cannot be ignored. This chemical along with others, which showed promising inhibitory activity against particular bacterial strains, should be subjected to further experiments to evaluate their efficacy and this will be the subject of our future research works.

### Antifungal Activity

**Fig. 2:**
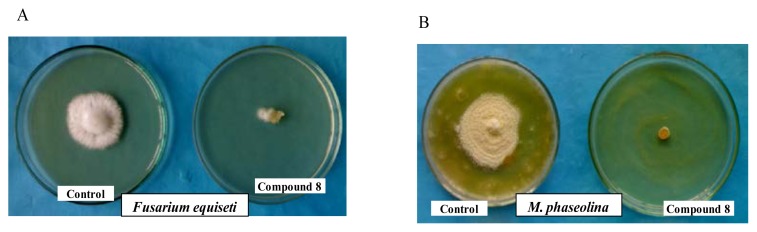
% Inhibition of mycelial growth against A: *F. equiseti* and B: *M. phaseolina* by compound **8**.

The results obtained from the present investigation of the antifungal studies as mentioned in Table 5 and Figure 2 clearly demonstrated that compound **8** showed the highest inhibition against all of these fungal strains. Here the percent inhibition of compound **8** is higher than the standard antibiotic, Nystatin in all the cases. The rest of the compounds was moderate or less sensitive towards the five tested fungal phytopathogens. The antifungal activities of our test compounds are in accordance with the results we observed before [24–25]. From the MIC test results reported in Table 6, it was observed that the extract exhibited a varied range of MIC values from 62.5 μg/mL to 250 μg/mL. The lowest MIC (62.5 μg/mL) was recorded against the pathogenic plant fungi, *M. phaseolina* and *C. corchori.*

Therefore, it is expected that this work employing carbohydrate derivatives as test compounds will help further work for the development of pesticides and medicine for human disease control. So it is hoped that the acylated derivatives (Scheme 1 and Scheme 1) might show potential antiviral, antituberculatic, and anti-inflammatory activities.

### Statistical Analysis

The standard deviation value is expressed in terms of ±SD. On the basis of the calculated value by using the ANOVA method, it has been observed that the differences below the 0.0001 level (*p* ≤ 0.0001) were considered as statistically significant.

## Experimental

### Materials and Methods

The ^1^H-NMR (400 MHz) and ^13^C-NMR (100 MHz) spectra were recorded for solutions in deuteriochloroform (CDCl_3_) using tetramethylsilane (TMS) as internal standard with a Bruker DPX-400 spectrometer at the Bangladesh Council of Scientific and Industrial Research (BCSIR) Laboratories, Dhaka, Bangladesh. Evaporations were carried out under reduced pressure using a VV-1 type vacuum rotary evaporator (Germany) with a bath temperature below 40°C. Melting points were determined on an electro-thermal melting point apparatus (England) and are uncorrected. All reagents used were commercially available (Aldrich) and were used as received, unless otherwise specified. Column chromatography was performed with silica gel G_60_. The solvent system employed for the TLC analyses was ethyl acetate-hexane in different proportions. Thin layer chromategraphy (TLC) was performed on Kieselgel GF_254_ and the spots were detected by spraying the plates with 1% H_2_SO_4_ and heating at 150–200°C until coloration took place. The reaction pathways have been summarized in Schemes 1 and 2.

### Synthesis of methyl 2,6-di-O-hexanoyl-α-D-glucopyranoside (2)

A solution of methyl α-D-glucopyranoside (**1**) (5 g, 25.75 mmol) in dry pyridine (60 mL) was cooled to −5°C whereupon hexanoyl chloride (3.9 mL, 28.97 mmol) was added to it. The mixture was stirred at the same temperature for 4 hours and then stirred overnight at room temperature. The progress of the reaction was monitored by TLC, which indicated the formation of two products, the slower-moving component being the major one. A few pieces of ice were added to the flask and then the product mixture was extracted with chloroform (3×10 mL). The combined chloroform layer was washed successively with dilute hydrochloric acid (10%), saturated aqueous sodium hydrogen carbonate (NaHCO_3_) solution, and distilled water. The chloroform layer was dried (MgSO_4_), filtered, and the filtrate was concentrated under reduced pressure to leave a syrup. The syrup was passed through a silica gel column and eluted with methanol-chloroform (1:20). Initial elution provided the faster-moving component which could not be isolated in pure form. Further elution furnished the 2,6-di-*O*-hexanoyl derivative (**2**).

Yield: 45%, Syrup, R*_f_* = 0.51 (CH_3_OH/CHCl_3_, 1/20). IR (KBr, cm^−1^): 1730 (C=O), 3610 (-OH). ^1^H-NMR (400 MHz, CDCl_3_, TMS): δ=4.85 (1H, d, *J*=3.6 Hz, H-1), 4.64 (1H, dd, *J*=3.7 and 10.0 Hz, H-2), 4.38 (1H, dd, *J*=5.0 and 12.1 Hz, H-6a), 4.27 (1H, dd, *J*=2.0 and 12.1 Hz, H-6b), 3.90 (1H, t, *J*=9.3 Hz, H-3), 3.72 (1H, t, *J*=9.7 Hz, H-4), 3.71 (1H, ddd, *J*=2.9, 9.9 and 12.8 Hz, H-5), 3.33 (3H, s, 1-OC*H*_3_), 2.35 {2H, t, *J*=7.5 Hz, CH_3_(CH_2_)_3_C*H*_2_CO-}, 2.33 {2H, t, *J*=7.5 Hz, CH_3_(CH_2_)_3_C*H*_2_CO-}, 1.60 {4H, m, 2×CH_3_(CH_2_)_2_C*H*_2_CH_2_CO-}, 1.27 {8H, m, 2×CH_3_(C*H*_2_)_2_(CH_2_)_2_CO-}, 0.85 {6H, t, *J*=6.7 Hz, 2×C*H*_3_(CH_2_)_4_CO-}. ^13^C-NMR (100 MHz): δ=174.51, 173.76 {2×CH_3_(CH_2_)_4_*C*O*-*}, 97.09 (C-1), 72.92 (C-2), 71.32 (C-4), 70.62 (C-3), 69.38 (C-5), 63.05 (C-6), 55.16 (1-O*C*H_3_), 34.09, 34.05, 31.21, 31.12, 24.53, 24.44, 22.27, 22.22 {2×CH_3_(*C*H_2_)_4_CO*-*}, 13.83, 13.81 {2×*C*H_3_(CH_2_)_4_CO*-*}; ^13^C-DEPT (100 MHz): δ=Upside signals: 97.09 (C-1), 72.92 (C-2), 71.32 (C-4), 70.62 (C-3), 69.38 (C-5), 55.16 (1-O*C*H_3_), 13.83, 13.81 {2 × *C*H_3_(CH_2_)_4_CO*-*}, Downside signals: 63.05 (C-6), 34.09, 34.05, 31.21, 31.12, 24.53, 24.44, 22.27, 22.22 {2×CH_3_(*C*H_2_)_4_CO}. Anal. calcd. for C_19_H_32_O_8_: C, 58.75; H, 8.29%; Found: C, 58.79; H, 8.44%.

### General Procedure of the Synthesis of Compounds (3–9)

A stirred and cooled (0°C) solution of the 3,4-diol (**2**) (100 mg, 0.25 mmol) in dry C_5_H_5_N (3 mL) was separately treated with Ac_2_O (0.16 mL, 1.71 mmol), methanesulphonyl chloride (0.15 mL, 1.93 mmol), 4-methoxybenzoyl chloride (0.3 mL, 1.76 mmol), 4-nitrobenzoyl chloride (223 mg, 0.44 mmol), 4-chlorobenzoyl chloride (0.22 mL, 1.74 mmol), 3-chlorobenzoyl chloride (0.21 mL, 1.66 mmol), and pentanoyl chloride (0.21 mL, 1.73mmol), respectively, and stirring was continued at 0°C for 6 hours. TLC examination (ethyl acetate-*n*-hexane, 1:4) showed the complete conversion of reactant into a single product. Excess reagent was destroyed by the addition of a few pieces of ice and the reaction mixture was extracted with chloroform (3×10 mL). The combined organic extract was washed successively with dilute hydrochloric acid, saturated aqueous sodium hydrogen carbonate solution, and water. The organic layer was dried (MgSO_4_), filtered, and the filtrate was evaporated off. The resulting syrupy residue was passed through silica gel column chromatography and eluted with ethyl acetate-*n*-hexane to afford compounds 3,4-di-*O*-acetyl derivative (**3**), **4**, **5**, **6**, **7**, **8,** and **9**, respectively.

#### Methyl 3,4-di-O-acetyl-2,6-di-O-hexanoyl-α-D-glucopyranoside (**3**)

Yield 92%, Thick syrup, R*_f_* = 0.51 (EtOAc/*n*-C_6_H_6_, 1/4). IR (KBr, cm^−1^): 1710 (C=O). ^1^H-NMR (400 MHz, CDCl_3_, TMS): δ=5.45 (1H, t, *J*= 9.8 Hz, H-3), 5.02 (1H, t, *J*=9.9 Hz, H-4), 4.92 (1H, d, *J*=3.6 Hz, H-1), 4.85 (1H, dd, *J*=3.6 and 10.2 Hz, H-2), 4.21 (1H, dd, *J*=4.8 and 12.3 Hz, H-6a), 4.11 (1H, dd, *J*=2.2 and 12.2 Hz, H-6b), 3.96 (1H, m, H-5), 3.37 (3H, s, 1-OC*H*_3_), 2.28 {4H, m, 2×CH_3_(CH_2_)_3_C*H*_2_CO-}, 1.99, 1.96 {2×3H, 2×s, 2×C*H*_3_CO-}, 1.59 {4H, m, 2×CH_3_(CH_2_)_2_C*H*_2_CH_2_CO-}, 1.27 {8H, m, 2×CH_3_(C*H*_2_)_2_(CH_2_)_2_CO-}, 0.86 {6H, m, 2×C*H*_3_(CH_2_)_4_CO-}. ^13^C-NMR (100 MHz): δ=173.42, 172.96 {2×CH_3_(CH_2_)_4_*C*O*-*}, 169.95, 169.51 {2×CH_3_*C*O*-*}, 96.81 (C-1), 70.63 (C-2), 70.14 (C-4), 68.67 (C-3), 67.23 (C-5), 61.79 (C-6), 55.41 (1-O*C*H_3_), 34.03, 33.99, 31.24, 31.10, 24.60, 24.46, 22.27, 22.21 {2×CH_3_(*C*H_2_)_4_CO*-*}, 20.63, 20.58 {2×*C*H_3_CO*-*}, 13.86, 13.83 {2×*C*H_3_(CH_2_)_4_CO*-*}; ^13^C-DEPT (100 MHz): δ=Upside signals: 96.82 (C-1), 70.63 (C-2), 70.14 (C-4), 68.68 (C-3), 67.23 (C-5), 55.41 (1-O*C*H_3_), 20.63, 20.58 {2×*C*H_3_CO*-*}, 13.86, 13.83 {2×*C*H_3_(CH_2_)_4_CO*-*}, Downside signals: 61.79 (C-6), 34.03, 33.99, 31.24, 31.10, 24.60, 24.46, 22.27, 22.22 {2×CH_3_(*C*H_2_)_4_CO*-*}. Anal. calcd. for C_23_H_36_O_10_: C, 58.46; H, 7.66%; Found: C, 58.48; H, 7.65%.

#### Methyl 2,6-di-O-hexanoyl-3,4-bis-O-(methylsulfonyl)-α-D-glucopyranoside (**4**)

Yield 95%, Syrupy, R*_f_* = 0.52 (EtOAc/*n*-C_6_H_6_, 1/2); IR (KBr, cm^−1^): 1720 (C=O). ^1^H-NMR (400 MHz, CDCl_3_, TMS): δ=5.10 (1H, t, *J*=9.4 Hz, H-3), 4.92 (1H, d, *J*=3.6Hz, H-1), 4.91 (1H, dd, *J*=3.6 and 10.2 Hz, H-2), 4.80 (1H, t, *J*=9.6 Hz, H-4), 4.35 (2H, m, H-6a and H-6b), 3.97 (1H, m, H-5), 3.37 (3H, s, 1-OC*H*_3_), 3.14, 3.10 {2×3H, 2×s, 2×C*H*_3_SO_2_-}, 2.36 {4H, m, 2×CH_3_(CH_2_)_3_C*H*_2_CO-}, 1.63 {4H, m, 2×CH_3_(CH_2_)_2_C*H*_2_*C*H_2_CO-}, 1.27 {8H, m, 2×CH_3_ (C*H*_2_)_2_(CH_2_)_2_CO-}, 0.87 {6H, m, 2×C*H*_3_(CH_2_)_4_CO-}. ^13^C-NMR (100 MHz): δ=173.34, 172.73 {2×CH_3_(CH_2_)_4_*C*O*-*}, 96.76 (C-1), 76.46 (C-2), 73.13 (C-4), 70.29 (C-3), 67.31 (C-5), 61.50 (C-6), 39.08, 38.97 (2×*C*H_3_SO_2_-), 33.95, 33.85, 31.23, 31.12, 24.42, 24.20, 22.26, 22.24 {2×CH_3_(*C*H_2_)_4_CO*-*}, 13.86 (×2) {2×*C*H_3_(CH_2_)_4_CO*-*}; ^13^C-DEPT(100 MHz): δ=Upside signals: 96.76 (C-1), 76.47 (C-2), 73.13 (C-4), 70.29 (C-3), 67.31 (C-5), 55.69 (1-O*C*H_3_), 39.08, 38.97 {2×*C*H_3_SO_2_*-*}, 13.86 (×2) {2×*C*H_3_(CH_2_)_4_CO*-*}, Downside signals: 61.50 (C-6), 33.95, 33.85, 31.23, 31.12, 24.42, 24.20, 22.27, 22.24 {2×CH_3_(*C*H_2_)_4_CO*-*}; Anal. calcd. for C_21_H_36_S_2_O_12_: C, 46.32; H, 6.25%; Found: C, 46.77; H, 6.31%.

#### Methyl 2,6-di-O-hexanoyl-3,4-di-O-(4-methoxybenzoyl)-α-D-glucopyranoside (**5**)

Yield 81%, Semi-solid, R_f_ = 0.55 (EtOAc/n-C_6_H_6_, 1/3); IR (KBr, cm^−1^): 1690 (C=O). ^1^H-NMR (400 MHz, CDCl_3_): δ=8.07 (2H, d, J=8.4 Hz, Ar-H), 7.84 (2H, t, J=8.1 Hz, Ar-H), 6.96 (2H, d, J=8.4 Hz, Ar-H), 6.80 (2H, t, J=7.5 Hz, Ar-H), 5.89 (1H, t, J=9.8 Hz, H-3), 5.42 (1H, t, J=9.6 Hz, H-4), 5.11(1H, m, H-6a), 5.01 (1H, d, J=3.6Hz, H-1), 4.22 (1H, m, H-6b), 4.19 (1H, m, H-5), 3.87 (3H, s, Ar-OC*H*_3_), 3.41 (3H, s, 1-OC*H*_3_), 2.30, 2.22 {2×2H, 2×m, 2×CH_3_(CH_2_)_3_C*H*_2_CO-},1.59, 1.42 {2×2H, 2×m, 2×CH_3_(CH_2_)_2_C*H*_2_CH_2_CO-}, 1.26 {8H, m, 2×CH_3_(C*H*_2_)_2_(CH_2_)_2_CO-}, 0.86 {6H, m, 2×C*H*_3_(CH_2_)_4_CO-}. ^13^C-NMR (100 MHz): δ=173.39, 173.10 {2×CH_3_(CH_2_)_4_ *C*O*-*}, 164.90, 162.26 (2×4-OCH_3_.C_6_H_4_.*C*O-), 132.79 (×4), 131.92, 131.80, 114.13 (×4), 113.65, 113.56 (2×4-OCH_3._*C*_6_H_4_CO-), 97.12 (C-1), 70.86 (C-2), 70.00 (C-4), 69.23 (C-3), 67.69 (C-5), 62.42 (C-6), 55.55 (1-O*C*H_3_), 55.43, 55.36 (2×4-O*C*H_3._C_6_H_4_.CO-), 34.05, 33.97, 31.23, 30.97, 24.49, 24.45, 22.26, 22.08 {2×CH_3_(*C*H_2_)_4_CO*-*}, 13.86, 13.66 {2×*C*H_3_(CH_2_)_4_CO*-*}; ^13^C-DEPT (100 MHz): δ Upside signals: 97.12 (C-1), 70.86 (C-2), 70.00 (C-4), 69.23(C-3), 67.69 (C-5), 13.86, 13.66 { 2×*C*H_3_(CH_2_)_4_CO*-*}, Downside signals: 62.42 (C-6), 34.05, 33.97, 31.22, 30.97, 24.49, 24.44, 22.26, 22.08 {2×CH_3_(*C*H_2_)_4_CO*-*}; Anal. calcd. for C_35_H_44_O_10_: C, 67.30; H, 7.08%; Found: C, 67.09; H, 7.04%.

#### Methyl 2,6-di-O-hexanoyl-3,4-bis-O-(4-nitrobenzoyl)-α-D-glucopyranoside (**6**)

Yield 88%, Syrup, R*_f_* = 0.58 (EtOAc/*n*-C_6_H_6_, 1/3); IR (KBr, cm^−1^): 1722 (C=O). ^1^H-NMR (400 MHz, CDCl_3_, TMS): δ=8.19 (4H, m, Ar-H), 8.05 (4H, m, Ar-H), 5.92 (1H, t, *J*=9.8 Hz, H-3), 5.48 (1H, t, *J*=9.7 Hz, H-4), 5.18 (1H, dd, *J*=3.6 and 10.3 Hz, H-2), 5.03 (1H, d, *J*=3.6 Hz, H-1), 4.29 (2H, m, H-6a and H-6b), 4.23 (1H, m, H-5), 3.48 (3H, s, 1-OC*H*_3_), 2.33, 2.24 {2×2H, 2×m, 2×CH_3_(CH_2_)_3_C*H*_2_CO-},1.61, 1.43 {2×2H, 2×m, 2×CH_3_(CH_2_)_2_C*H*_2_CH_2_CO-}, 1.26, 1.09 {2×4H, 2×m, 2×CH_3_(C*H*_2_)_2_(CH_2_)_2_CO-}, 0.86, 0.70 {2×3H, 2×t, 2×C*H*_3_(CH_2_)_4_CO-}. ^13^C-NMR (100 MHz): δ=173.29, 172.88 {2×CH_3_(CH_2_)_4_*C*O*-*}, 163.79, 163.45 (2×4- NO_2._C_6_H_4_.*C*O-), 150.86, 150.81, 134.18, 134.01, 130.90 (×2), 130.83 (×2), 123.64 (×2), 123.61 (×2) (2×4-NO_2._*C*_6_H_4_CO-), 97.05 (C-1), 71.72 (C-2), 70.26 (C-4), 70.15 (C-3), 67.25 (C-5), 61.81 (C-6), 55.71 (1-O*C*H_3_), 33.94 (×2), 31.21, 30.95, 24.49, 24.43, 22.25, 22.06 {2×CH_3_(*C*H_2_)_4_CO*-*}, 13.85, 13.63 {2×*C*H_3_(CH_2_)_4_CO*-*}; ^13^C-DEPT (100 MHz): δ=Upside signals: 97.06 (C-1), 71.73 (C-2), 70.27 (C-4), 70.15(C-3), 67.26 (C-5), 55.71 (1-O*C*H_3_), 13.85, 13.63 { 2×*C*H_3_(CH_2_)_4_CO*-*}, Downside signals: 61.81 (C-6), 33.94 (×2), 31.21, 30.95, 24.49, 24.43, 22.25, 22.06 {2×CH_3_(*C*H_2_)_4_CO}; Anal. calcd. for C_33_H_38_N_2_O_14_: C, 57.72; H, 5.56%; Found: C, 57.78; H, 5.55%.

#### Methyl 3,4-bis-O-(4-chlorobenzoyl)-2,6-di-O-hexanoyl-α-D-glucopyranoside (**7**)

Yield 93%, Crystalline solid (recrystallization from EtOAc), mp. 134–135°C, R*_f_* = 0.54 (EtOAc/*n*-C_6_H_6_, 1/8); IR (KBr, cm^−1^): 1710 (C=O). ^1^H-NMR (400 MHz, CDCl_3_, TMS): δ=8.06 (2H, d, *J*=7.9 Hz, Ar-H), 8.02 (2H, d, *J*=8.0 Hz, Ar-H), 7.50 (2H, d, *J*=7.8 Hz, Ar-H), 7.44 (2H, d, *J*=7.9 Hz, Ar-H), 5.89 (1H, t, *J*=9.8 Hz, H-3), 5.52 (1H, t, *J*=9.6 Hz, H-4), 5.43 (1H, t, *J*=9.7 Hz, H-6a), 5.13 (1H, dd, *J*=3.0 and 9.6 Hz, H-2), 5.09 (1H, d, *J*=3.5 Hz, H-1), 4.22 (2H, m, H-5 and H-6b), 3.47 (3H, s, 1-OC*H*_3_), 2.38, 2.30 {2×2H, 2×m, 2×CH_3_(CH_2_)_3_C*H*_2_CO-}, 1.63, 1.44 {2×2H, 2×m, 2×CH_3_(CH_2_)_2_C*H*_2_CH_2_CO-}, 1.26 {8H, m, 2×CH_3_(C*H*_2_)_2_(CH_2_)_2_CO-}, 0.88, 0.73 {2×3H, 2×m, 2×C*H*_3_(CH_2_)_4_CO-}. **^1^**^3^C-NMR (100 MHz): δ=173.21, 173.15 {2×CH_3_(CH_2_)_4_*C*O*-*}, 165.38, 164.25 (2×4-Cl.C_6_H_4_.*C*O-), 131.91 (×3), 131.58, 131.13, 129.41 (×4), 128.92 (×3) (2×4-Cl.*C*_6_H_4_.CO-), 97.31 (C-1), 70.71 (C-2), 70.42 (C-4), 69.28 (C-3), 67.75 (C-5), 62.51 (C-6), 55.45 (1-O*C*H_3_), 34.17, 33.91, 31.29, 31.01, 24.64, 24.46, 22.32, 22.16 {2×CH_3_(*C*H_2_)_4_CO*-*}, 13.91, 13.75 {2×*C*H_3_(CH_2_)_4_CO*-*}; Anal. calcd. for C_33_H_38_O_10_Cl: C, 62.90; H, 6.07%; Found: C, 62.98; H, 6.11%.

#### Methyl 3,4-bis-O-(3-chlorobenzoyl)-2,6-di-O-hexanoyl-α-D-glucopyranoside (**8**)

Yield 87%, Pasty mass, R*_f_* = 0.56 (EtOAc/*n*-C_6_H_6_, 1/6); IR (KBr, cm^−1^): 1715 (C=O). ^1^H-NMR (400 MHz, CDCl_3_, TMS): δ=8.06, 8.00, 7.60, 7.46 (4×2H, 4×m, 2×3-Cl.C_6_*H*_4_CO-), 5.92 (1H, t, *J*=9.8 Hz, H-3), 5.47 (1H, t, *J*=9.5 Hz, H-4), 5.14 (1H, dd, *J*=3.6 and 10.2 Hz, H-2), 5.03 (1H, d, *J*=3.6 Hz, H-1), 4.22 (3H, m, H-5, H-6a and H-6b), 3.47 (3H, s, 1-OC*H*_3_), 2.32, 2.22 {2×2H, 2×m, 2×CH_3_(CH_2_)_3_C*H*_2_CO}, 1.66, 1.59 {2×2H, 2×m, 2×CH_3_(CH_2_)_2_C*H*_2_CH_2_CO-}, 1.37, 1.26 {2×4H, 2×m, 2×CH_3_(C*H*_2_)_2_(CH_2_)_2_CO-}, 0.88 {6H, m, 2×C*H*_3_(CH_2_)_4_CO-}. ^13^C-NMR (100 MHz): δ=173.30, 172.91{2×CH_3_(CH_2_)_4_*C*O*-*}, 164.31, 163.95 (2×3-Cl.C_6_H_4_.*C*O-), 97.05 (C-1), 70.91 (C-2), 70.54 (C-4), 69.72 (C-3), 67.30 (C-5), 62.05 (C-6), 55.49 (1-O*C*H_3_), 33.94, 33.87, 31.14, 30.88, 24.44, 24.37, 22.58, 22.18 {2×CH_3_(*C*H_2_)_4_CO*-*}, 13.93, 13.78 {2×*C*H_3_(CH_2_)_4_CO*-*}; ^13^C-DEPT (100 MHz): δ=Upside signals: 97.05 (C-1), 70.98 (C-2), 70.61 (C-4), 69.80 (C-3), 67.37 (C-5), 55.56 (1-O*C*H_3_), 13.85, 13.63 { 2×*C*H_3_(CH_2_)_4_CO*-*}, Downside signals: 62.12 (C-6), 34.01, 33.94, 31.21, 30.95, 24.51, 24.44, 22.65, 22.25 {2×CH_3_(*C*H_2_)_4_CO*-*}; Anal. calcd. for C_35_H_38_O_10_Cl: C, 64.27; H, 5.84%; Found: C, 64.69; H, 5.75%.

#### Methyl 2,6-di-O-hexanoyl-3,4-di-O-pentanoyl-α-D-glucopyranoside (**9**)

Yield 86%, Thick syrup, R*_f_* = 0.61 (EtOAc/*n*-C_6_H_6_, 1/6); IR (KBr, cm^−1^): 1721 (C=O). ^1^H-NMR (400 MHz, CDCl_3_, TMS): δ=5.46 (1H, t, *J*= 9.8 Hz, H-3), 5.03 (1H, t, *J*=9.9Hz, H-4), 4.90 (1H, d, *J*=3.6 Hz, H-1), 4.83 (1H, dd, *J*=3.6 and 10.2 Hz, H-2), 4.16 (1H, dd, *J*=4.8 and 12.2 Hz, H-6a), 4.08 (1H, dd, *J*=2.0 and 12.2 Hz, H-6b), 3.93 (1H, m, H-5), 3.35 (3H, s, 1-OC*H*_3_), 2.29, 2.19 {2×4H, 2×m, 2×CH_3_(CH_2_)_3_C*H*_2_CO- and 2×CH_3_(CH_2_)_2_C*H*_2_CO-},1.58, 1.48 {2×4H, 2×m, 2×CH_3_(CH_2_)_2_C*H*_2_CH_2_CO- and 2×CH_3_CH_2_CH_2_C*H*_2_CO-}, 1.24 {12H, m, 2×CH_3_(C*H*_2_)_2_(CH_2_)_2_CO- and 2×CH_3_C*H*_2_(CH_2_)_2_CO-}, 0.84 {12H, m, 2×C*H*_3_(CH_2_)_4_CO- and 2×C*H*_3_(CH_2_)_3_CO-}. ^13^C-NMR (100 MHz): δ=173.32, 172.87, 172.54, 172.16 {2×CH_3_(CH_2_)_4_*C*O*-* and 2×CH_3_(CH_2_)_3_*C*O*-*}, 96.80 (C-1), 70.75 (C-2), 69.65 (C-4), 68.26 (C-3), 67.32 (C-5), 61.78 (C-6), 55.32 (1-O*C*H_3_), 33.93 (×2), 33.80, 33.65, 31.19, 31.09, 26.86, 26.71, 24.48, 24.41, 22.22, 22.17, 22.12 (×2) {2×CH_3_(*C*H_2_)_4_CO*-* and 2×CH_3_(*C*H_2_)_3_CO*-*}, 13.80, 13.77, 13.52 (×2) {2×*C*H_3_(CH_2_)_4_CO*-* and 2×*C*H_3_(CH_2_)_3_CO*-*}; ^13^C-DEPT (100 MHz): δ=Upside signals: 96.80 (C-1), 70.74 (C-2), 69.65 (C-4), 68.25 (C-3), 67.32 (C-5), 55.32 (1-O*C*H_3_), 13.79, 13.76, 13.52 (×2) {2×*C*H_3_(CH_2_)_4_CO*-* and 2×*C*H_3_(CH_2_)_3_CO*-*}, Downside signals: 61.77 (C-6), 33.93 (×2), 33.80, 33.64, 31.19, 31.08, 26.86, 26.70, 24.48, 24.41, 22.22, 22.16, 22.12 (×2){2×CH_3_(*C*H_2_)_4_CO*-* and 2×CH_3_(*C*H_2_)_3_CO*-*}; Anal. calcd. for C_29_H_48_O_10_: C, 62.57; H, 8.67%; Found: C, 62.77; H, 8.74%.

### General Procedure for the Synthesis of Compounds 10 and 11

A cooled (0°C) and stirred solution of methyl 3-*O*-benzoyl-4,6-*O*-benzylidene-α-D-mannopyranoside (**1A**) [26] (100 mg, 0.26 mmol) in anhydrous pyridine (3 mL) was allowed to react with pentanoyl chloride (0.07 mL, 0.56 mmol) and stirring was continued for 8 hours. TLC examination indicated the formation of a faster-moving product. A few pieces of ice were added to the flask with constant shaking and the mixture was extracted three times with chloroform. The combined chloroform extract was washed successively with dilute hydrochloric acid, saturated aqueous sodium hydrogen carbonate solution, and distilled water. The organic layer was dried (Na_2_SO_4_), filtered, and concentrated. Purification of the resulting syrupy residue was effected by silica gel column chromatography (ethyl acetate-hexane as eluant) to furnish the title compound (**10**). A similar reaction and purification procedure was applied to prepare compound **11**.

#### Methyl 3-O-benzoyl-4,6-O-benzylidene-2-O-pentanoyl-α-D-mannopyranoside (**10**)

Yield 90%, Thick syrup, R*_f_* = 0.51 (EtOAc/*n*-C_6_H_6_, 1/9); IR (KBr, cm^−1^): 1718 (C=O). ^1^H-NMR data (400 MHz, CDCl_3_, TMS): δ=7.98 (2H, d, *J*=7.6 Hz, Ar-H), 7.52 (1H, t, *J*=7.0 Hz, Ar-H), 7.41 (4H, m, Ar-H), 7.31 (3H, m, Ar-H), 5.66 (1H, dd, *J*=3.5 and 10.3 Hz, H-3), 5.62 (1H, s, C_6_H_5_C*H*-), 5.48(1H, d, *J*=3.5 Hz, H-2), 4.72 (1H, s, H-1), 4.33 (1H, dd, *J*=4.6 and 10.1 Hz, H-6a), 4.20 (1H, t, *J*=9.6 Hz, H-6b), 4.02 (1H, ddd, *J*=4.7, 10.0 and 10.0 Hz, H-5), 3.90 (1H, t, *J*=10.2 Hz, H-4), 3.43 (3H, s, 1-OC*H*_3_), 2.41 {2H, t, *J*=7.4 Hz, CH_3_(CH_2_)_2_C*H*_2_CO-}, 1.59 {2H, m, 2× CH_3_CH_2_C*H*_2_CH_2_CO-}, 1.32 {2H, m, CH_3_C*H*_2_(CH_2_)_2_CO-}, 0.86 {3H, m, C*H*_3_(CH_2_)_3_CO-}. ^13^C-NMR (100 MHz): δ=172.52 {CH_3_(CH_2_)_3_*C*O*-*}, 165.29 (C_6_H_5_*C*O), 137.06, 133.01, 129.70 (×2), 129.65, 128.96 (×2), 128.33, 128.20, 128.14, 126.07 (×2) (*C*_6_H_5_CH- and *C*_6_H_5_CO-), 101.77 (C_6_H_5_*C*H-), 99.57 (C-1), 76.43(C-4), 69.98 (C-3), 68.84 (C-2), 68.75 (C-5), 60.32 (C-6), 55.18 (1-O*C*H_3_), 33.85, 26.98, 22.11 {CH_3_(*C*H_2_)_3_CO*-*}, 13.62 {*C*H_3_(CH_2_)_3_CO*-*}; Anal. calcd. for C_26_H_35_O_8_: C, 65.67; H, 7.40%; Found: C, 65.88; H, 7.55%.

#### Methyl 3-O-benzoyl-4,6-O-benzylidene-2-O-hexanoyl-α-D-mannopyranoside (**11**)

Yield 92%, Syrupy, R_f_ = 0.51 (EtOAc/n-C_6_H_6_, 1/6); IR (KBr, cm^−1^): 1708 (C=O). ^1^H-NMR (400 MHz, CDCl_3_, TMS): δ_H_ 7.98 (2H, d, *J*= 7.6 Hz, Ar-H), 7.52 (1H, t, *J*=7.3 Hz, Ar-H), 7.40 (4H, m, Ar-H), 7.31 (3H, m, Ar-H), 5.66 (1H, dd, *J*=3.2 and 10.1 Hz, H-3), 5.62 (1H, s, C_6_H_5_C*H*-), 5.48(1H, d, *J*=3.2 Hz, H-2), 4.72 (1H, s, H-1), 4.32 (1H, dd, *J*=4.5 and 10.1 Hz, H-6a), 4.19 (1H, t, *J*=9.7 Hz, H-6b), 4.03 (1H, ddd, *J*=4.4, 9.9 and 10.0 Hz, H-5), 3.89 (1H, t, *J*=10.2 Hz, H-4), 3.43 (3H, s, 1-OC*H*_3_), 2.40 {2H, t, *J*=7.4 Hz, CH_3_(CH_2_)_3_C*H*_2_CO-},1.60 {2H, m, CH_3_(CH_2_)_2_C*H*_2_CH_2_CO-}, 1.27 {4H, m, CH_3_(C*H*_2_)_2_(CH_2_)_2_CO-}, 0.86 {3H, m, C*H*_3_(CH_2_)_4_CO-}. ^13^C-NMR (100 MHz): δ=172.59 {CH_3_(CH_2_)_4_*C*O*-*}, 165.34, (C_6_H_5_*C*O-), 137.09, 133.06, 129.70 (×3), 129.02, 128.26 (×3), 126.11 (×3) (*C*_6_H_5_CH- and *C*_6_H_5_CO-), 101.83 (C_6_H_5_*C*H-), 99.63 (C-1), 76.49 (C-4), 70.03 (C-3), 68.90 (C-2), 68.82 (C-5), 63.74 (C-6), 55.25 (1-O*C*H_3_), 34.15, 31.18, 24.62, 22.27 {CH_3_(*C*H_2_)_4_CO*-*}, 13.84 {*C*H_3_(CH_2_)_4_CO*-*}; Anal. calcd. for C_27_H_27_O_8_: C, 67.63; H, 5.66%; Found: C, 67.79; H, 5.74%.

### General Procedure for the Synthesis of Compounds (12–14)

An ice-cooled solution of methyl 4,6-*O*-benzylidene-2-*O*-(3,5-dinitrobenzoyl)-α-D-mannopyranoside (**1B**) [27] (100 mg, 0.21 mmol) in dry pyridine (6 mL) was treated with pentanoyl chloride (0.052 mL, 0.42 mmol) and the solution was stirred at this temperature for 4 hours and then at room temperature for 12 hours. TLC examination indicated the full conversion of the starting material into a single product. Excess reagent was destroyed by the addition of a few pieces of ice and the reaction mixture was processed as usual. The resulting syrup was purified by column chromatography (with ethyl acetate-hexane as eluant) to afford the pentanoyl derivative (**12**). Recrystalization from ethyl acetate-hexane gave compound **12**. A similar reaction and purification was used to isolate compounds **13**, **14,** and **15**.

#### Methyl 4,6-O-benzylidene-2-O-(3,5-dinitrobenzoyl)-3-O-pentanoyl-α-D-mannopyranoside (**12**)

Yield 91%, Crystalline solid as needles, mp. 142–143°C, R*_f_* = 0.53 (EtOAc/*n*-C_6_H_6_, 1/6); IR (KBr, cm^−1^): 1711 (C=O). ^1^H-NMR (400 MHz, CDCl_3_, TMS): δ=9.24 (1H, s, Ar-H), 9.14 (2H, d, *J*=1.9 Hz, Ar-H), 7.44 (2H, d, *J*=3.9 Hz, Ar-H), 7.35 (3H, t, *J*=3.8 Hz, Ar-H), 5.79 (1H, t, *J*=9.5 Hz, H-3), 5.54 (1H, s, C_6_H_5_C*H*-), 5.14 (1H, d, *J*=3.9 Hz, H-1), 5.12 (1H, dd, *J*=3.9 and 9.5 Hz, H-2), 4.35 (1H, dd, *J*=4.8 and 10.2 Hz, H-6a), 3.99 (1H, ddd, *J*=5.0, 10.0 and 10.2 Hz, H-5), 3.84 (1H, t, *J*=9.4 Hz, H-6b), 3.75 (1H, t, *J*=9.6 Hz, H-4), 3.42 (3H, s, 1-OC*H*_3_), 2.27 {2H, m, CH_3_(CH_2_)_2_C*H*_2_CO-}, 1.47 {2H, t, *J*=7.5 Hz, CH_3_CH_2_C*H*_2_CH_2_CO-}, 1.16 {2H, m, CH_3_C*H*_2_(CH_2_)_2_CO-}, 0.69 {3H, t, *J*=7.3 Hz, C*H*_3_(CH_2_)_3_CO-}. ^13^C-NMR (100 MHz): δ=172.75 {CH_3_(CH_2_)_3_*C*O*-*}, 162.07 (3,5(NO_2_)_2_.C_6_H_3_*C*O-), 148.79 (×2), 136.77, 129.77 (×2), 128.27 (×3), 126.17 (×3), 122.85 {*C*_6_H_5_CH- and 3,5(NO_2_)_2_.*C*_6_H_3_CO-}, 101.73 (C_6_H_5_*C*H-), 97.14 (C-1), 76.72 (C-4), 74.32 (C-3), 68.76 (×2) (C-2 and C-5), 62.43 (C-6), 55.46 (1-O*C*H_3_), 33.95, 27.03, 22.02 {CH_3_(*C*H_2_)_3_CO*-*}, 13.52 {*C*H_3_(CH_2_)_3_CO*-*}; Anal. calcd. for C_26_H_28_N_2_O_12_: C, 57.14; H, 5.15%; Found: C, 57.79; H, 5.45.

#### Methyl 4,6-O-benzylidene-2-O-(3,5-dinitrobenzoyl)-3-O-hexanoyl-α-D-mannopyranoside (**13**)

Yield 82%, Crystalline solid (recrystallization from EtOAc), mp. 137–138°C, R*_f_* = 0.53 (EtOAc/*n*-C_6_H_6_, 1/4); IR (KBr, cm^−1^): 1712 (C=O). ^1^H-NMR (400 MHz, CDCl_3_, TMS): δ=9.23 (1H, s, Ar-H), 9.14 (2H, d, *J*=1.7 Hz, Ar-H), 7.44 (2H, d, *J*=3.9 Hz, Ar-H), 7.35 (3H, t, *J*=3.7 Hz, Ar-H), 5.79 (1H, t, *J*=9.4 Hz, H-3), 5.54 (1H, s, C_6_H_5_C*H*-), 5.14 (1H, d, *J*=3.8 Hz, H-1), 5.12 (1H, dd, *J*=3.8 and 9.4 Hz, H-2), 4.35 (1H, dd, *J*=4.7 and 10.2 Hz, H-6a), 4.00 (1H, ddd, *J*= 4.9, 9.9 and 10.2 Hz, H-5),3.82 (1H, t, *J*=10.3 Hz, H-6b), 3.76 (1H, t, *J*=9.7 Hz, H-4), 3.42 (3H, s, 1-OC*H*_3_), 2.26 {2H, m, CH_3_(CH_2_)_3_C*H*_2_CO-}, 1.49 {2H, t, *J*=7.1 Hz, CH_3_(CH_2_)_2_C*H*_2_CH_2_CO-}, 1.11 {4H, m, CH_3_(C*H*_2_)_2_(CH_2_)_2_CO-}, 0.67 {3H, t, *J*=6.7 Hz, C*H*_3_(CH_2_)_4_CO-}. ^13^C-NMR (100 MHz): δ=172.80 {CH_3_(CH_2_)_4_*C*O*-*}, 162.04 {3,5(NO_2_)_2_.C_6_H_3_*C*O-}, 148.78 (×2), 136.79, 129.77 (×2), 129.18, 128.26 (×3), 126.15 (×3) {*C*_6_H_5_CH- and 3,5(NO_2_)_2_.*C*_6_H_3_CO-}, 101.72 (C_6_H_5_*C*H-), 97.16 (C-1), 76.71 (C-4), 74.30 (C-3), 68.76 (×2) (C-2 and C-5), 62.42 (C-6), 55.45 (1-O*C*H_3_), 34.19, 31.02, 24.66, 22.14 {CH_3_(*C*H_2_)_4_CO*-*}, 13.68 {*C*H_3_(CH_2_)_4_CO*-*}; Anal. calcd. for C_27_H_30_N_2_O_12_: C, 57.85; H, 5.38%; Found: C, 57.66; H, 5.33%.

#### Methyl 4,6-O-benzylidene-2-O-(3,5-dinitrobenzoyl)-3-O-(4-methoxybenzoyl)-α-D-mannopyranoside (**14**)

Yield 85%, Syrupy mass, R_f_ = 0.50 (EtOAc/n-C_6_H_6_, 1/3); IR (KBr, cm^−1^): 1716 (C=O). ^1^H-NMR (400 MHz, CDCl_3_, TMS): δ=9.13 (1H, s, Ar-H), 9.07 (2H, d, *J*=2.0 Hz, Ar-H), 8.08 (2H, d, *J*=8.8 Hz, Ar-H), 7.42 (3H, m, Ar-H), 7.30 (2H, m, Ar-H), 6.97 (2H, d, *J*=8.8 Hz, Ar-H), 6.04 (1H, t, *J*=9.5 Hz, H-3), 5.58 (1H, s, C_6_H_5_C*H*-), 5.20 (2H, m, H-1 and H-2), 4.38 (1H, dd, *J*=4.7 and 10.4 Hz, H-6a), 4.08 (2H, m, H-5 and H-6b), 3.92 (1H, t, *J*=9.6 Hz, H-4), 3.88 (3H, s, 4-OC*H*_3_.C_6_H_4_CO-), 3.45 (3H, s, 1-OC*H*_3_). ^13^C-NMR (100 MHz): δ=164.60, 162.29 {3,5(NO_2_)_2_.C_6_H_3_*C*O- and 4-OCH_3_.C_6_H_4_*C*O-}, 132.82 (×5), 132.31, 131.92, 129.74, 128.21, 126.21, 121.33(×2), 114.15 (×5), 113.76 {*C*_6_H_5_CH-, 3,5(NO_2_)_2_.*C*_6_H_3_CO- and 4-OCH_3_.*C*_6_H_4_CO-}, 101.75 (C_6_H_5_*C*H-), 97.21 (C-1), 77.58 (C-4), 74.42 (C-3), 70.15 (C-2), 69.01 (C-5), 62.52 (C-6), 55.59 (4-O*C*H_3_.C_6_H_4_CO-), 55.48 (1-O*C*H_3_); Anal. calcd. for C_29_H_26_N_2_O_12_: C, 60.01; H, 4.50%; Found: C, 60.32; H, 4.53%.

### Antibacterial Screening Studies

#### Test Microorganisms

The synthesized test chemicals (**2–14**) were subjected to antimicrobial screening against ten human pathogenic and five phytopathogenic microorganisms. Test tube cultures of bacterial and fungal pathogens were obtained from the microbiology laboratory, Department of Microbiology, University of Chittagong, Bangladesh. The synthesized test compounds (schemes 1 and 2) were subjected to antibacterial screening against four Gram-positive and six Gram-negative bacterial strains viz., *Staphylococcus aureus* ATCC 6538, *Bacillus subtilis* BTCC 17, *Bacillus megaterium* BTCC 18, *Bacillus cereus* BTCC 19, *Shigella dysenteriae* AE 14396, *Shigella sonnei* CRL (ICDDR,B), *Salmonella typhi* AE 14612, *Salmonella paratyphi* AE 146313, *Pseudomonas* Species CRL (ICDDR,B), INABA ET (*Vibrio*) AE 14748. The name of phytopathogenic fungi viz., *Fusarim equiseti* (*Corda*) *Sacc*., *Macrophomina phaseolina* (Tassi) Goid, *Colletotrichum corchori* (Ikata Yoshida), *Curvularia lunata* (Wakker Becdijin), *Alternaria alternata* (Fr.) Kedissler. In all cases, a 2% solution (in CHCl_3_) of the chemicals was used.

#### Antibacterial Activity Assay

The *in vitro* antibacterial activities of the synthesized compounds were detected by the disk diffusion method [28] with little modification [29]. Sterilized paper discs of 4 mm in diameter and Petri dishes of 150 mm in diameter were used throughout the experiment. The autoclaved Mueller-Hinton agar medium, cooled to 45°C, was poured into sterilized Petri dishes to a depth of 3 to 4 mm and after solidification of the agar medium; the plates were transferred to an incubator at 37°C for 15 to 20 minutes to dry off the moisture that developed on the agar surface. The plates were inoculated with the standard bacterial suspensions (as McFarland 0.5 standard) followed by the spread plate method and allowed to dry for 3 to 5 minutes. Dried and sterilized filter paper discs were treated separately with 50 μg dry weight/disc from 2% solution (in CHCl_3_) of each test chemical using a micropipette, dried in air under aseptic condition, and were placed at equidistance in a circle on the seeded plate. A control plate was also maintained in each case without any test chemical. These plates were kept for 4–6 hours at low temperature (4–6°C) and the test chemicals diffused from the discs to the surrounding media by this time. The plates were then incubated at 35±2°C for 24 hours to allow maximum growth of the organisms. The antibacterial activity of the test agent was determined by measuring the mean diameter of the zones of inhibition in millimeters. Each experiment was repeated thrice. The standard antibiotic, ampicillin (BEXIMCO Pharm Bangladesh Ltd), was used as a positive control and compared with tested compounds under identical conditions. The MICs of the tested compounds were determined by the disk diffusion [28] method and broth macrodilution [30] method.

#### Antifungal Activity Assay

The *in vitro* antifungal activity of the acylated chemicals was done by the Poisons Food technique [31] with some modifications [29]. Two percent solution of the test chemical (in CHCl_3_) was mixed with sterilized melted Saburaud agar medium to obtain the desired concentration (2%) and this was poured into sterilized Petri dishes. At the center of each plate, 5 days-old fungal mycelial block (4 mm in diameter) was inoculated and incubated at 27°C. A control set was also maintained in each experiment. The linear mycelial growth of fungus was measured after 3–5 days of incubation. The percentage inhibition of radial mycelial growth of the test fungus was calculated as follows:

$$\text{I}=\frac{\text{C}-\text{T}}{\text{C}}\times 100$$

Where, I = Percentage of inhibition, C = Diameter of the fungal colony in the control (CHCl_3_), T = Diameter of the fungal colony in the treatment. All the results were compared with the standard antifungal antibiotic Nystatin (100 μg/mL medium, BEXIMCO Pharm Bangladesh Ltd.).

## Conclusion

In this paper we have explored the synthesis, characterization, and antibacterial screening studies of some acylated monosaccharide derivatives obtained from the direct acylation method. This method demonstrates a very simple and efficient method for the synthesis. Methyl 2,6-di-*O-*hexanoyl-3,4-di-*O*-methanesulphonyl-α-D-glucopyranoside (**4**) and methyl 3,4-di*-O*-(4-chlorobenzoyl)-2,6-di*-O*-hexanoyl-α-D-glucopyranoside (**7**) were found to be encouraging in terms of high selectivity and excellent yields as 95% and 93%, respectively. Thus, a good number of test compounds reported herein exhibited promising antibacterial activity. Methyl 3,4-di*-O*-(3-chlorobenzoyl)-2,6-di-*O*-hexanoyl-α-D-glucopyranoside (**8**) exhibited the highest antibacterial and antifungal activities against all of the tested microorganisms. So, this compound may be targeted for future studies for its usage as a broad-spectrum antibiotic. This piece of work, in our opinion, has created an opportunity for further work with these test compounds, ultimately leading to the development of new pesticides/medicines for human disease control with fewer environmental hazards.

## Supporting Information



## Figures and Tables

**Sch. 1:**
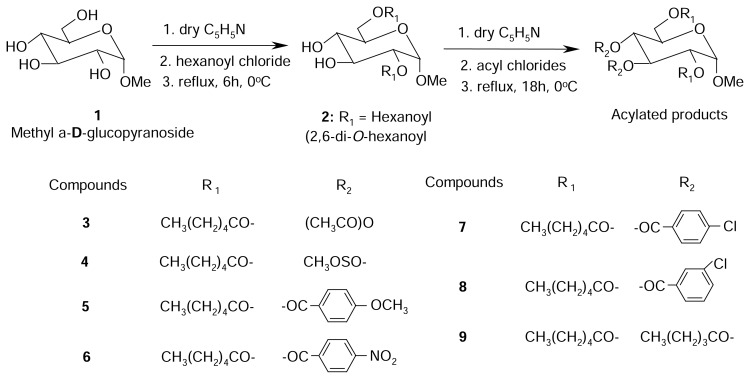
Synthesis of D-glucopyranoside derivatives (**2–9**).

**Sch. 2:**
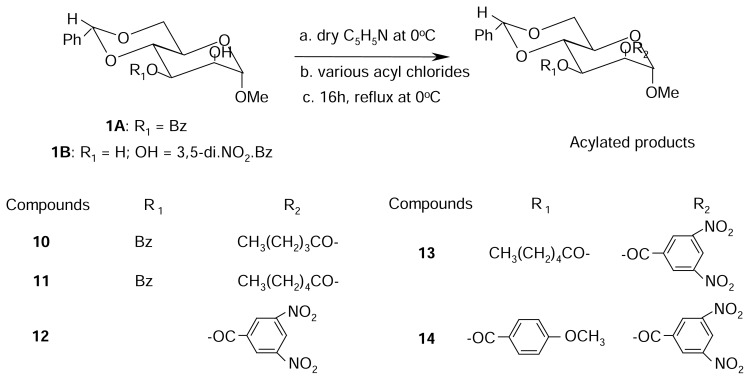
Synthesis of D-mannopyranoside derivatives (**10–14**).

**Tab. 1:** Antibacterial screening studies against Gram-positive bacteria by (**2–14**).

Compound	Diameter of zone of inhibition in mm 200 μg dw/disc			

	*B. subtilis*	*B. cereus*	*B. megaterium*	*S. aureus*
2	9	8	*20	11
3	NF	NF	NF	NF
4	NF	NF	NF	NF
5	7.5	6.5	12	10
6	NF	NF	NF	NF
7	14	14	10	12
8	*20	*26	*28	*27
9	NF	NF	NF	NF
10	NF	NF	NF	NF
11	NF	NF	NF	NF
12	NF	NF	NF	NF
13	NF	NF	NF	NF
14	7.5	NF	*20	6
**Ampicillin	*19	*18	*16	*22

* = marked inhibition;

** = standard antibiotic;

NF = not found; dw = dry weight.

**Tab. 2:** Antibacterial screening studies against Gram-negative bacteria of tested compounds (**2–14**).

Compound	Diameter of zone of inhibition in mm 200 μg dw/disc					

	*S. typhi*	*S. paratyphi*	*S. dysenteriae*	*S. sonnei*	*P.* species	INABAET *(Vibrio)*
2	20	11	19	11.5	7	7.5
3	NF	NF	NF	NF	NF	NF
4	NF	NF	NF	NF	NF	NF
5	10	7.5	9	8.5	6.5	7.5
6	NF	NF	NF	NF	NF	NF
7	16	11.5	8	15	NF	12
8	28	25	25	22	21	28
9	NF	NF	NF	NF	NF	NF
10	NF	NF	NF	NF	NF	NF
11	NF	NF	NF	NF	NF	NF
12	NF	NF	NF	NF	NF	NF
13	NF	NF	NF	NF	NF	NF
14	18	7.5	9	7.5	8	8
**Ampicillin	*20	*18	*22	*20	*18	*15

* = marked inhibition;

** = standard antibiotic;

NF = not found; dw = dry weight.

**Tab. 3:** MIC test of compound **8** against ten human pathogenic bacteria by disk diffusion method.

Test Bacteria	Diameter of zone of inhibition in mm							MIC μg/disc

	300 μg/disc	150 μg/disc	100 μg/disc	50 μg/disc	25 μg/disc	12.5 μg/disc	6.25 μg/disc	
*B. subtilis*	20	16	14	10	8	NF	NF	25
*B. cereus*	26	20	18	12	10	8	NF	12.5
*B. megaterium*	28	19	15	12	10	8	NF	12.5
*S. aureus*	27	20	15	11	10	8	NF	12.5
*P. species*	20	15	13	9	7	NF	NF	12.5
*S. typhi*	28	20	18	14	9	7.5	NF	12.5
*S. paratyphi*	25	15	14	11	8	NF	NF	25
*S. dysenteriae*	25	19	17	10	8	NF	NF	25
*S. sonnei*	22	17	13	10	8	NF	NF	25
INABAET (*Vibrio*)	28	20	18	14	10	8	NF	12.5

**Tab. 4:** MIC test of compound **8** against ten human pathogenic bacteria by broth macrodilution method.

Test Bacteria	Growth in peptone broth/extract concentration (μg/mL)							MIC μg/mL

	125	250	500	750	1000	1250	1500	
*B. subtilis*	+	+	+	+	+	−	−	1250
*B. cereus*	+	+	+	+	−	−	−	1000
*B. megaterium*	+	+	+	+	−	−	−	1000
*S. aureus*	+	+	+	+	−	−	−	1000
*P. species*	+	+	+	+	+	−	−	1250
*S. typhi*	+	+	+	+	+	−	−	1250
*S. paratyphi*	+	+	+	+	+	−	−	1250
*S. dysenteriae*	+	+	+	+	+	−	−	1250
*S. sonnei*	+	+	+	+	+	−	−	1250
INABAET (*Vibrio*)	+	+	+	+	−	−	−	1000

(+) indicate positive and (−) indicate negative.

**Tab. 5:** Antifungal activities of the test chemicals (**2–14**).

Compound	Inhibition % of fungal mycelial growth^a^ (100 μg dw/mL)				

	*F. equiseti*	*M. phaseolina*	*C. corchori*	*C. lunata*	*A. alternata*
2	30	20	25	25	30
3	25	40	10	20	22
4	25	12	18	15	15
5	34	36	25	30	35
6	10	NF	17	12	NF
7	40	35	30	40	35
8	*100	*97	*100	*100	*98
9	30	NF	9	NF	9
10	NF	NF	NF	10	NF
11	NF	16	12	15	10
12	NF	NF	NF	NF	NF
13	NF	NF	NF	8	NF
14	25	35	15	20	15
**Nystatin	*44.7	*71.78	*40.51	*75	*51.55

* = marked inhibition;

** = standard antibiotic;

NF = not found; dw = dry weight;

a = growth measured-radial in cm.

**Tab. 6:** MIC test of compound **8** against five phytopathogenic fungi.

Test Fungi	Growth in sabouroud/extract concentration (μg/mL)							MIC μg/mL

	31.25	62.5	125	250	500	750	1000	
*F. equiseti*	+	+	+	−	−	−	−	250
*A. alternata*	+	+	+	−	−	−	−	250
*M. phaseolina*	+	−	−	−	−	−	−	62.5
*C. corchori*	+	−	−	−	−	−	−	62.5
*C. lunata*	+	+	−	−	−	−	−	125

(+) indicate positive and (−) indicate negative.
