# Supplementary material for: Regioselective Synthesis, Characterization, and Antimicrobial Activities of Some New Monosaccharide Derivatives
Source: Sci Pharm. 2013 Sep 26;82(1):1–20. doi: 10.3797/scipharm.1308-03 (PMC3951222; doi:10.3797/scipharm.1308-03)
Supplement: Supplementary file 1 [file Scipharm.2014.82.1_supporting_information.pdf]

Scientia Pharmaceutica www.scipharm.at

Open Access

Supporting Information to

Regioselective Synthesis, Characterization, and Antimicrobial Activities of Some New Monosaccharide Derivatives

Sarkar M. A. KAWSAR, Md O. FARUK, Mohammad S. RAHMAN, Yuki FUJII, Yasuhiro OZEKI

Published in Sci Pharm. 2014; 82: 1–20 doi:10.3797/scipharm.1308-03

Available from: http://dx.doi.org/10.3797/scipharm.1308-03

© Kawsar *et al.*; licensee Österreichische Apotheker-Verlagsgesellschaft m. b. H., Vienna, Austria.

This is an Open Access article distributed under the terms of the Creative Commons Attribution License (http://creativecommons.org/licenses/by/3.0/), which permits unrestricted use, distribution, and reproduction in any medium, provided the original work is properly cited.

Table of Contents

Fig. S1. ^{1}H NMR spectrum (A) and expansion (B) of compound **2**.
Fig. S2. ^{13}C NMR spectrum of compound **2**.
Fig. S3. ^{1}H NMR spectrum (A) and expansion (B) of compound **3**.
Fig. S4. ^{13}C NMR spectrum of compound **3**.
Fig. S5. ^{1}H NMR spectrum (A) and expansion (B) of compound **4**.
Fig. S6. ^{13}C NMR spectrum of compound **4**.
Fig. S7. ^{1}H NMR spectrum (A) and expansion (B) of compound **5**.
Fig. S8. ^{13}C NMR spectrum of compound **5**.
Fig. S9. ^{1}H NMR spectrum (A) and expansion (B) of compound **6**.
Fig. S10. ^{13}C NMR spectrum of compound **6**.
Fig. S11. ^{1}H NMR spectrum (A) and expansion (B) of compound **8**.
Fig. S12. ^{13}C NMR spectrum of compound **8**.
Fig. S13. ^{1}H NMR spectrum (A) and expansion (B) of compound **9**.
Fig. S14. ^{13}C NMR spectrum of compound **9**.
Fig. S15. ^{1}H NMR spectrum (A) and expansion (B) of compound **10**.
Fig. S16. ^{13}C NMR spectrum of compound **10**.
Fig. S17. ^{1}H NMR spectrum of compound **11**.
Fig. S18. ^{13}C NMR spectrum of compound **11**.
Fig. S19. ^{1}H NMR spectrum (A) and expansion (B) of compound **12**.
Fig. S20. ^{1}H NMR spectrum of compound **13**.
Fig. S21. ^{13}C NMR spectrum of compound **13**.

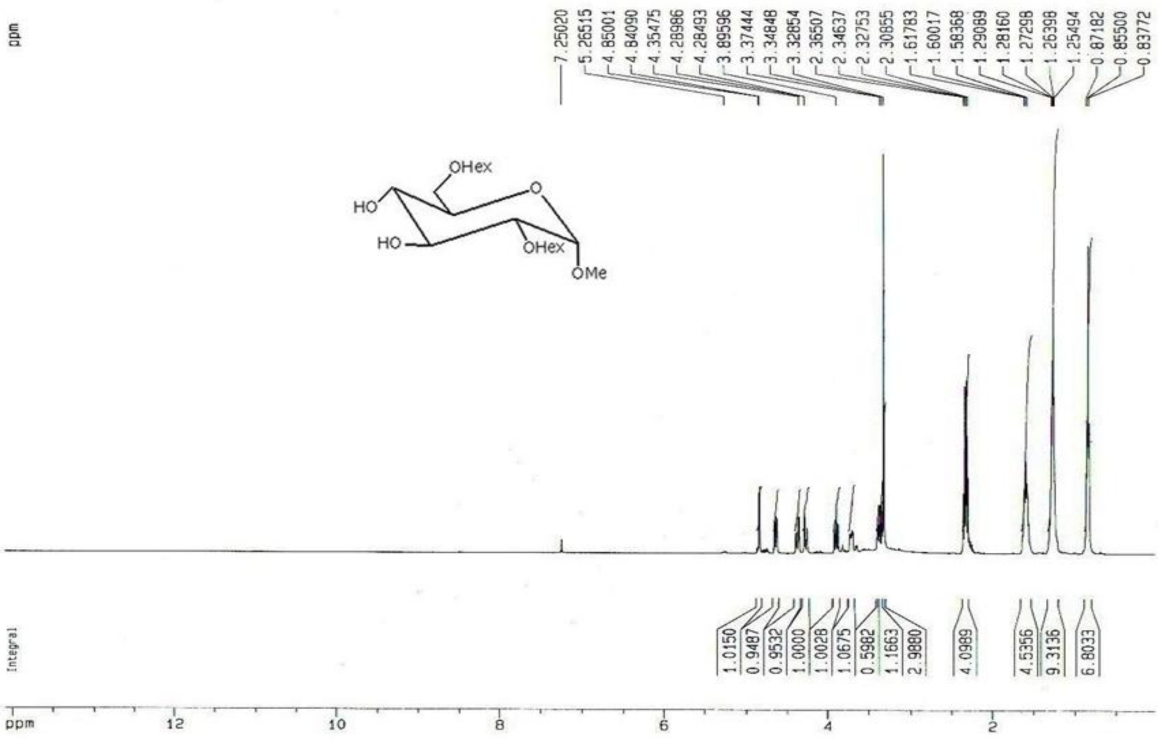

Fig. S1(A). ^{1}H NMR spectrum of compound **2**.

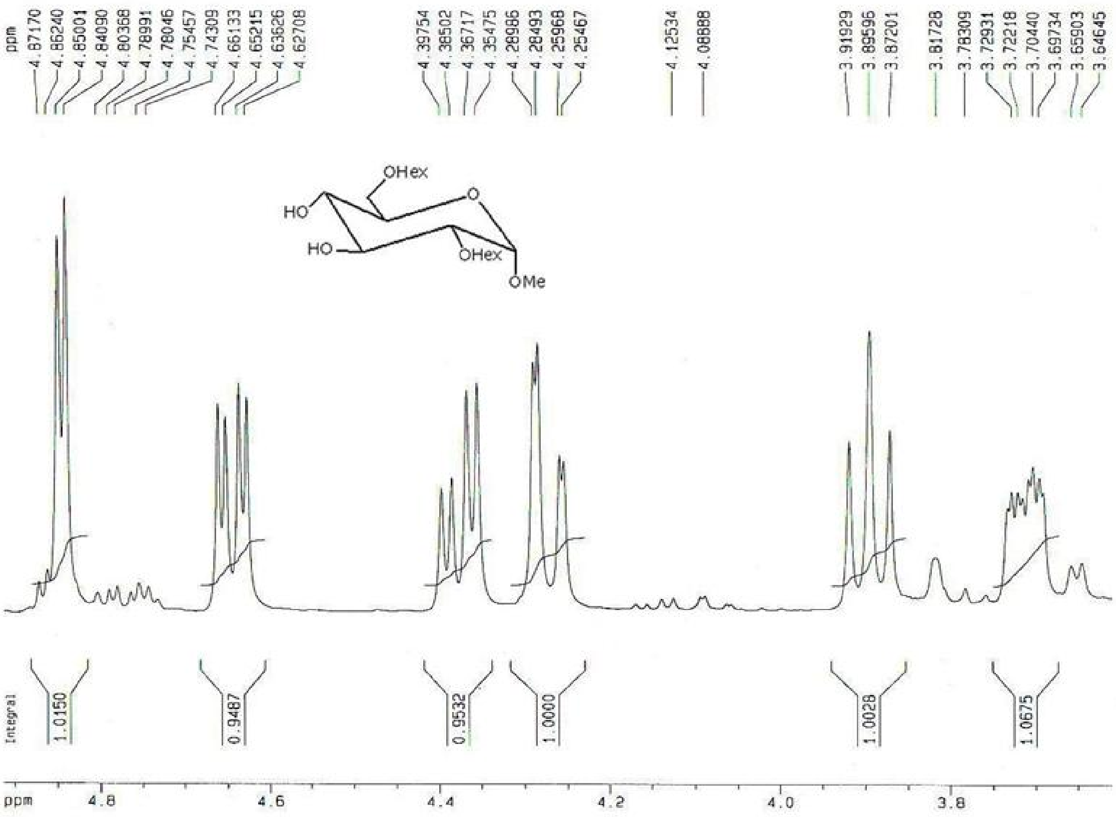

Fig. S1(B). ^{1}H NMR expansion of compound **2**.

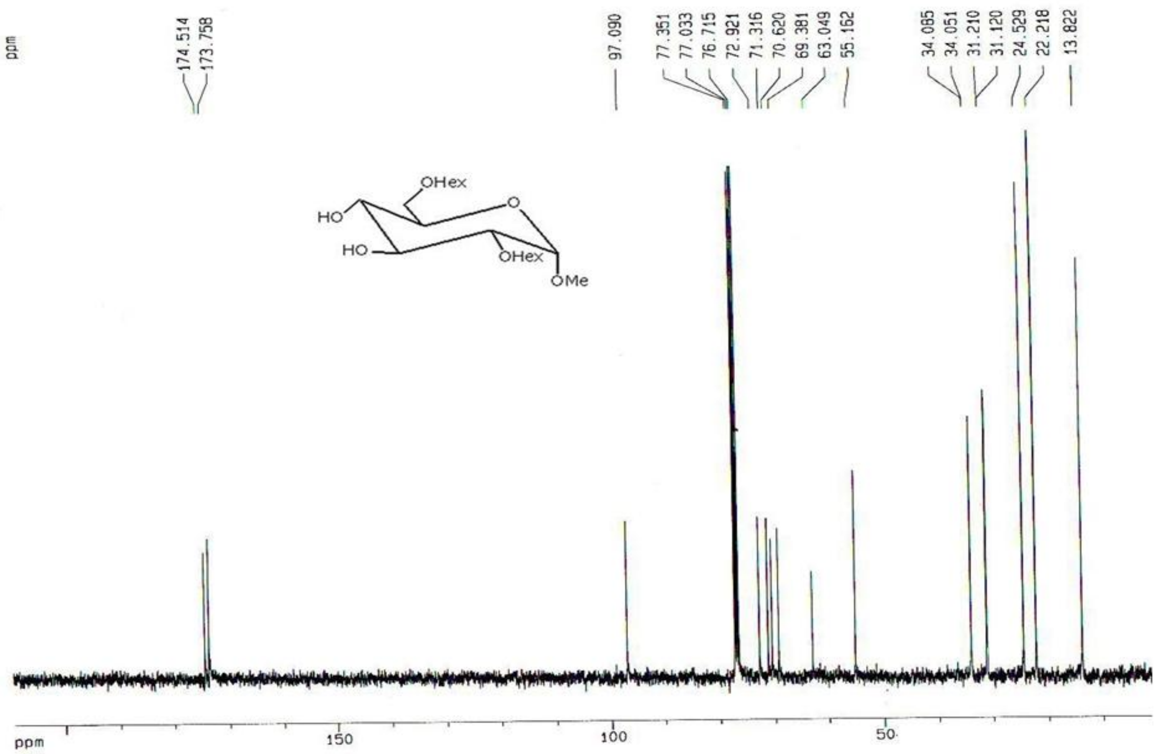

Fig. S2. ^{13}C NMR spectrum of compound **2**.

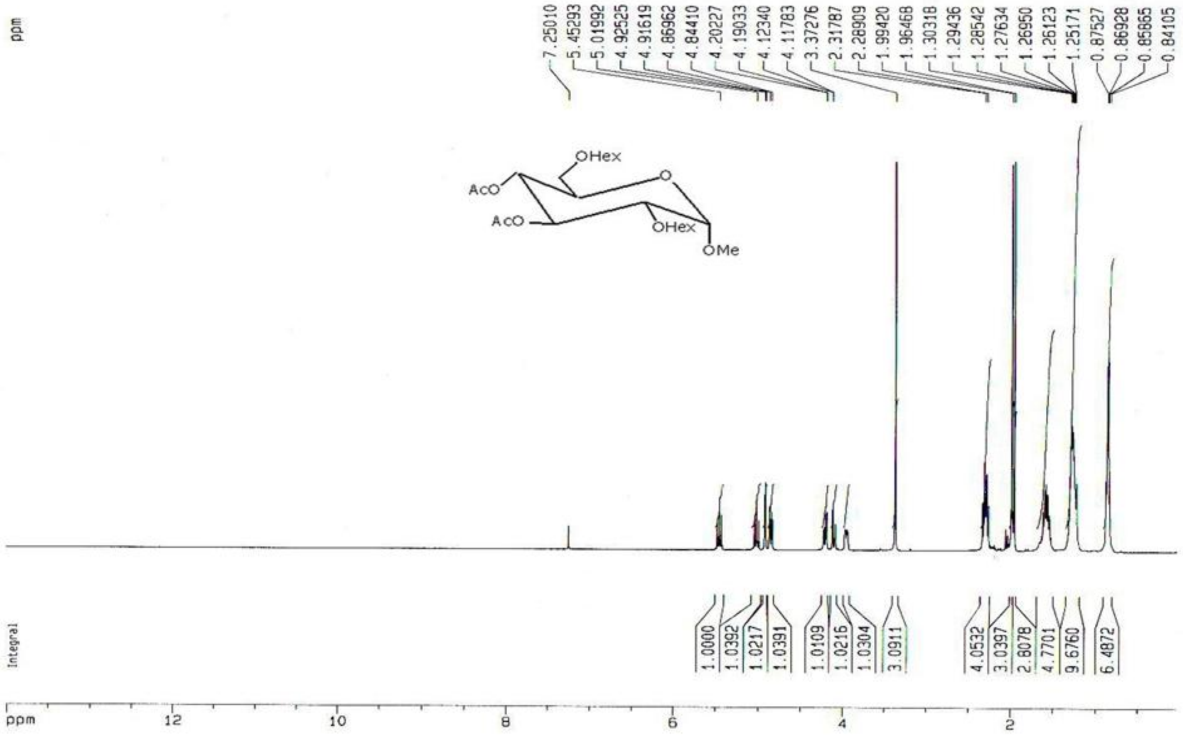

Fig. S3(A). ^{1}H NMR spectrum of compound **3**.

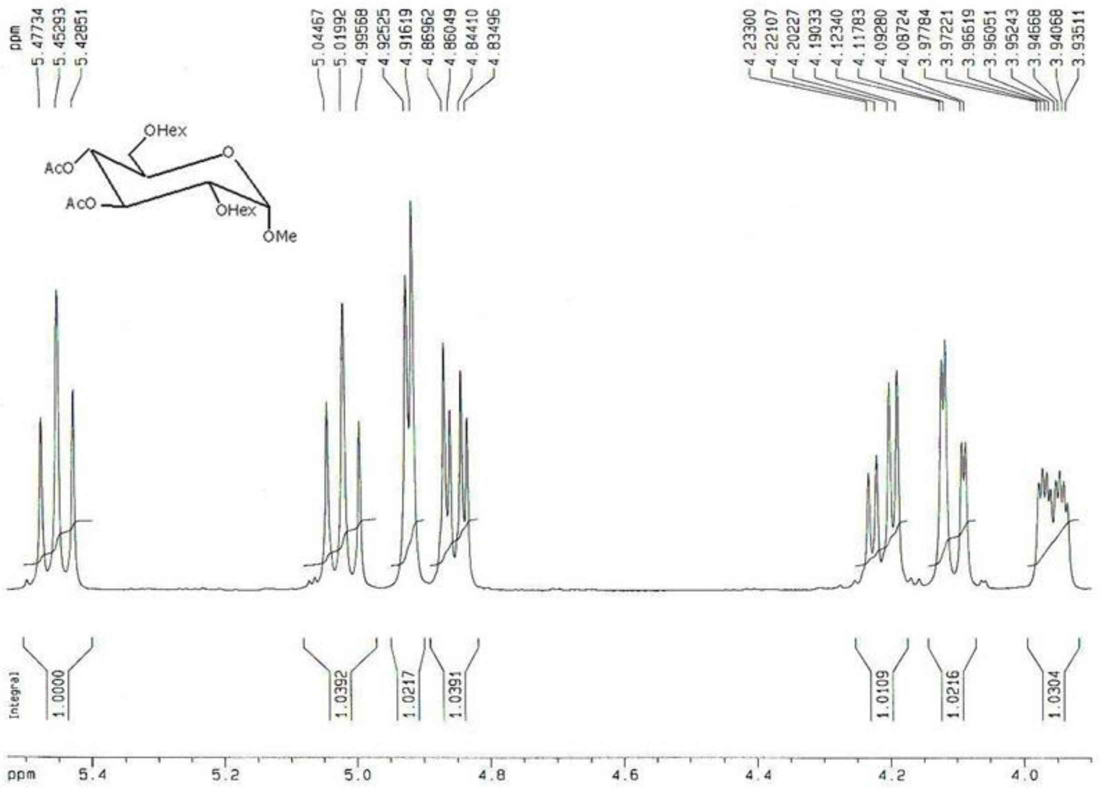

Fig. S3(B). ^{1}H NMR expansion of compound **3**.

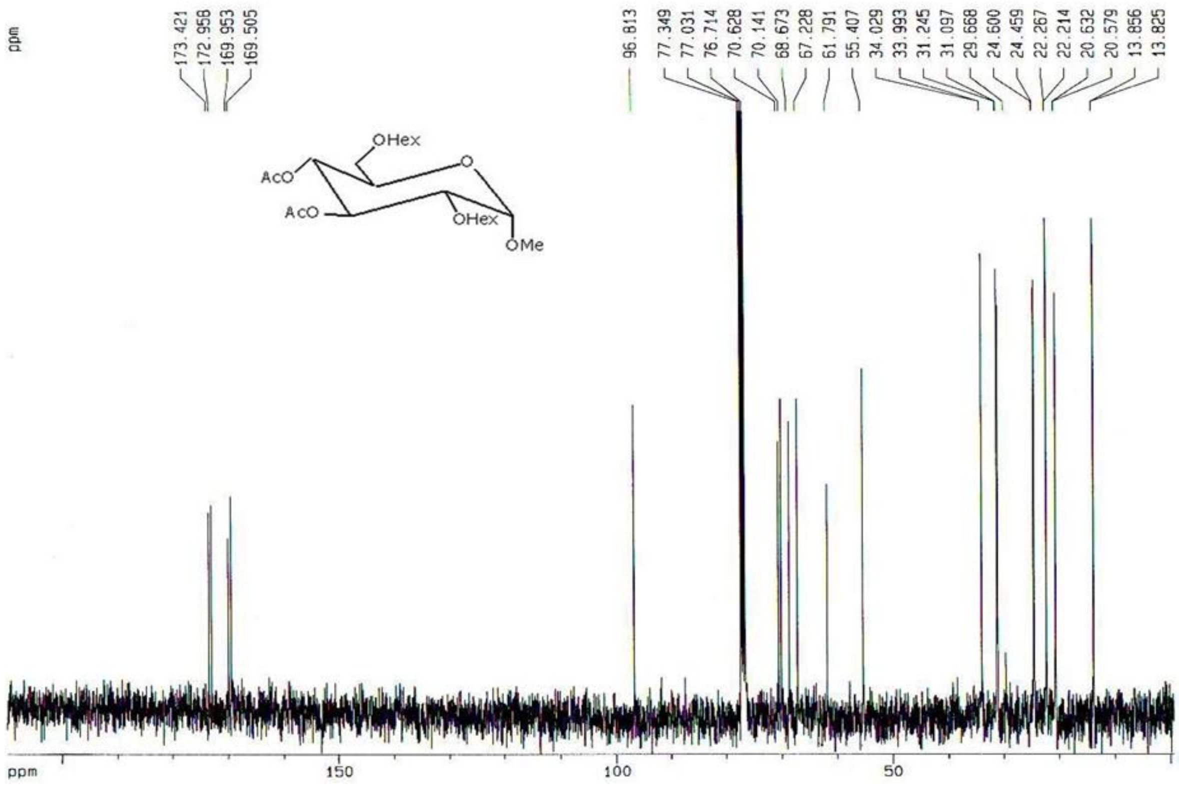

Fig. S4. ^{13}C NMR spectrum of compound **3**.

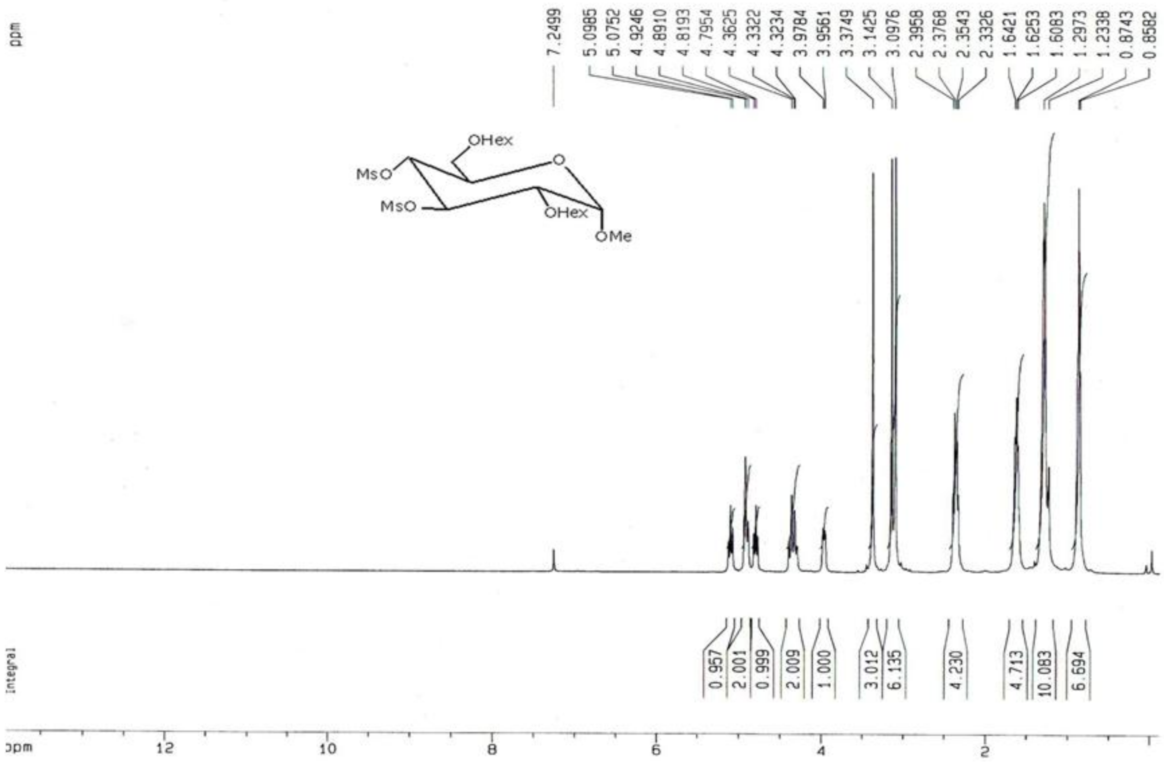

Fig. S5(A). ^{1}H NMR spectrum of compound **4**.

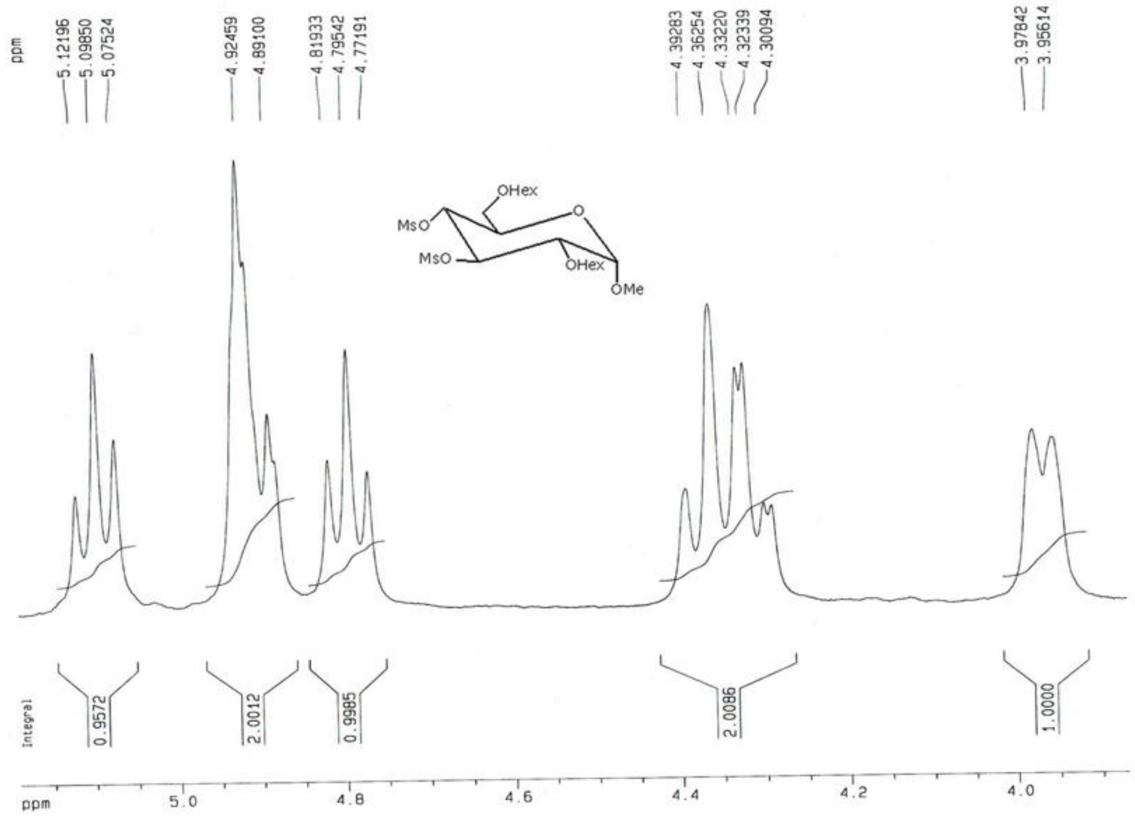

Fig. S5(B). ^{1}H NMR expansion of compound **4**.

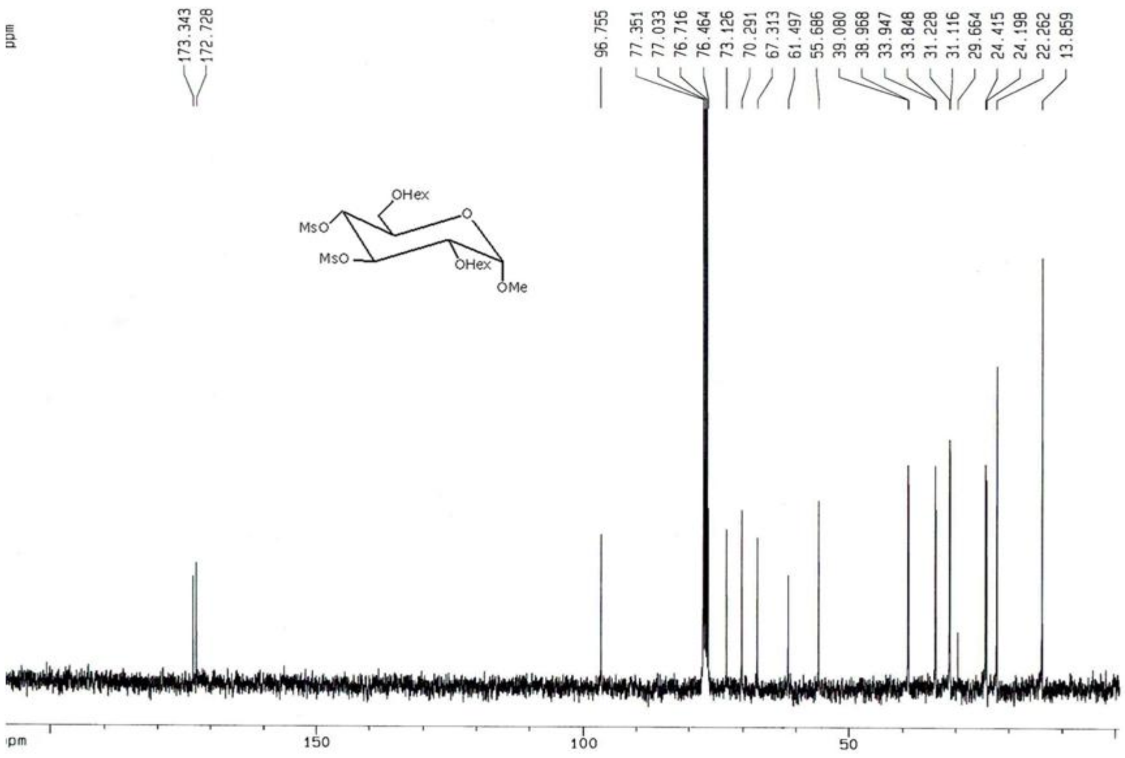

Fig. S6. ^{13}C NMR spectrum of compound **4**.

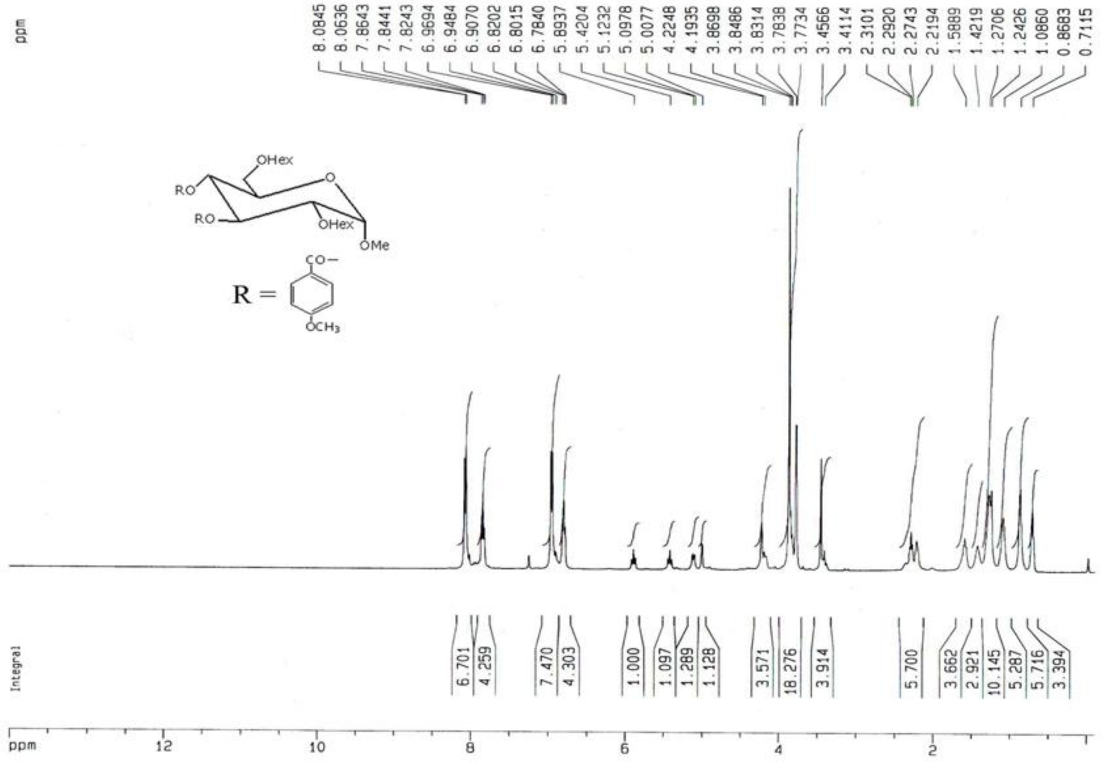

Fig. S7(A). ^{1}H NMR spectrum of compound **5**.

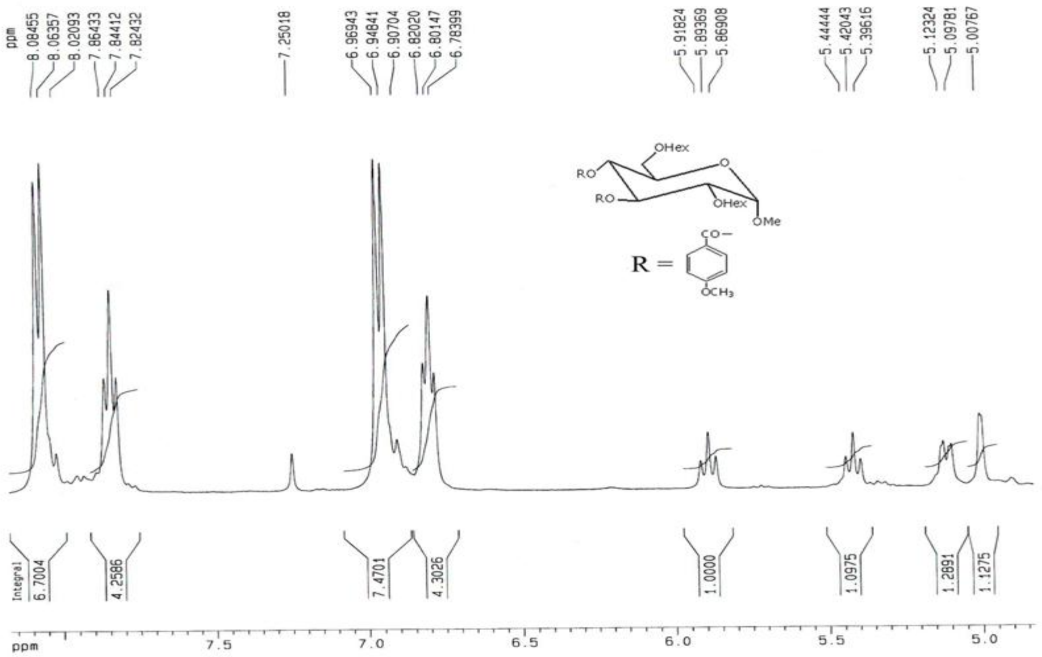

Fig. S7(B). ^{1}H NMR expansion of compound **5**.

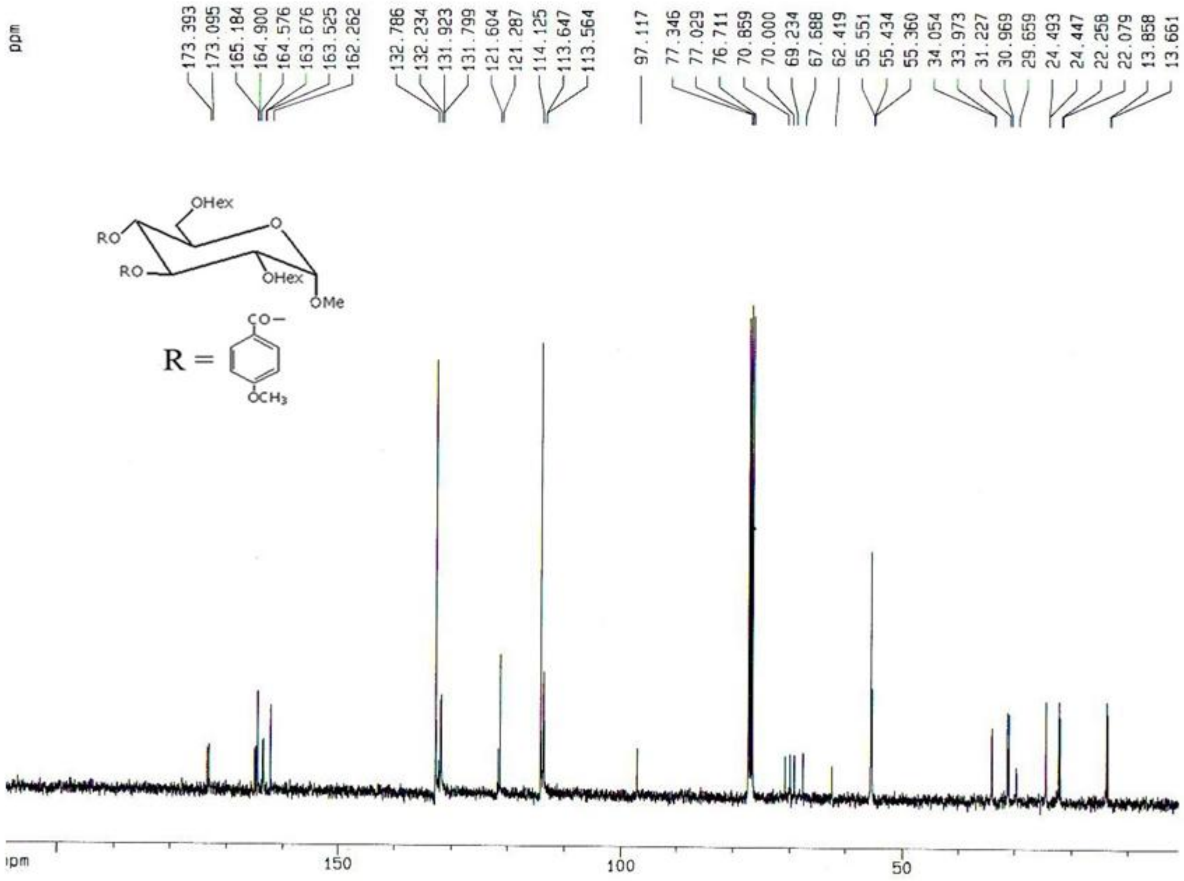

Fig. S8. ^{13}C NMR spectrum of compound **5**.

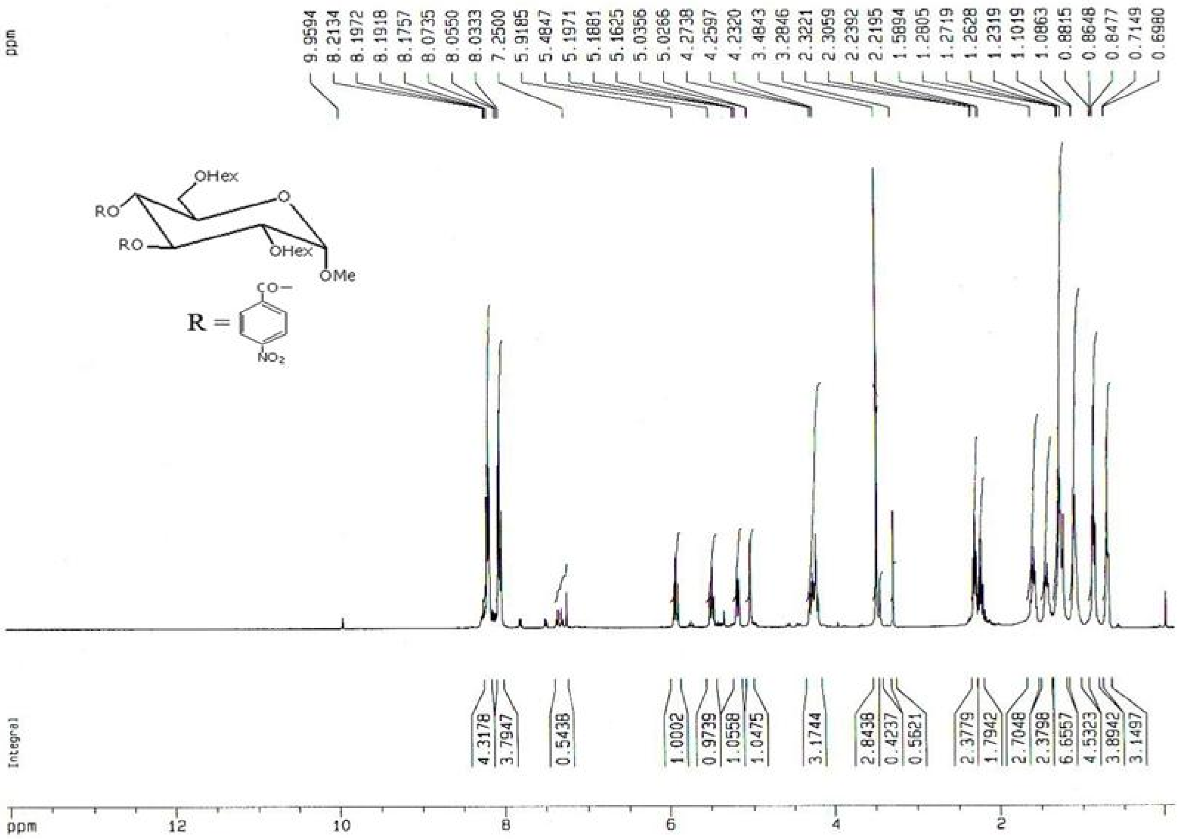

Fig. S9(A). ^{1}H NMR spectrum of compound **6**.

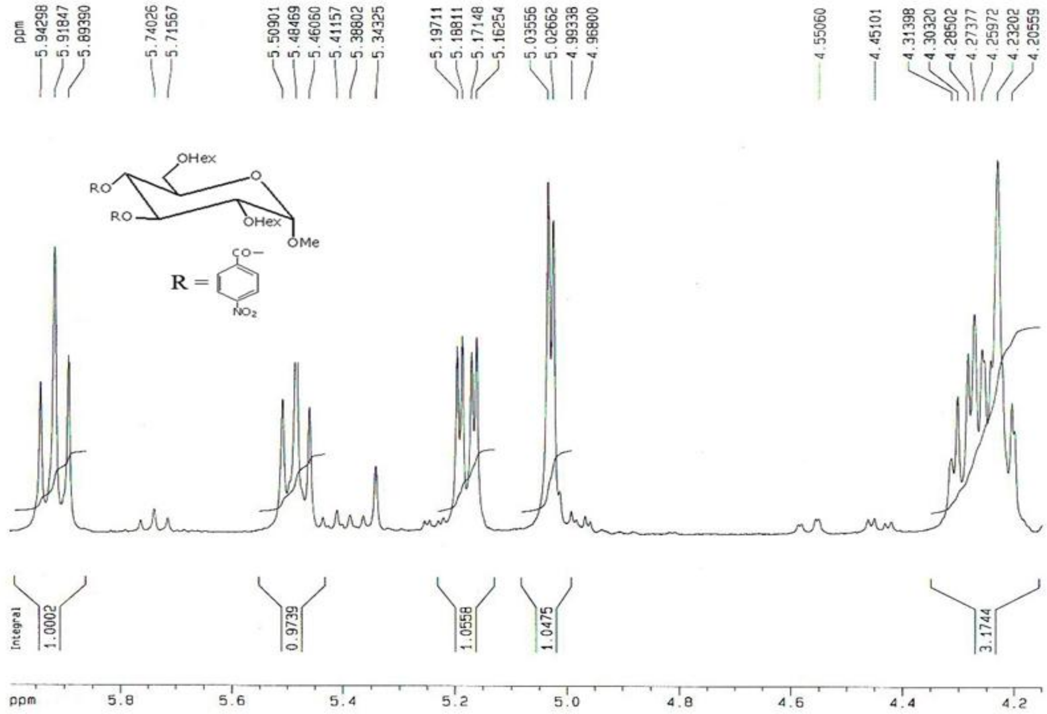

Fig. S9(B). ^{1}H NMR expansion of compound **6**.

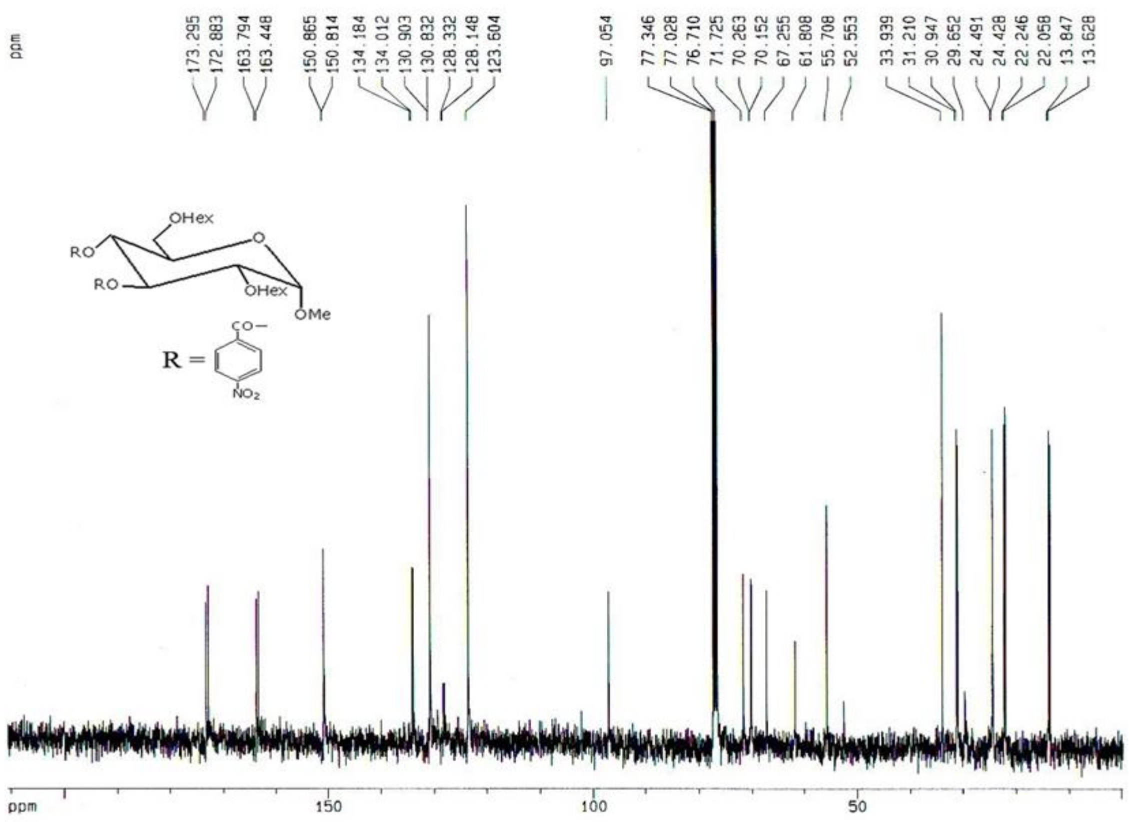

Fig. S10. ^{13}C NMR spectrum of compound **6**.

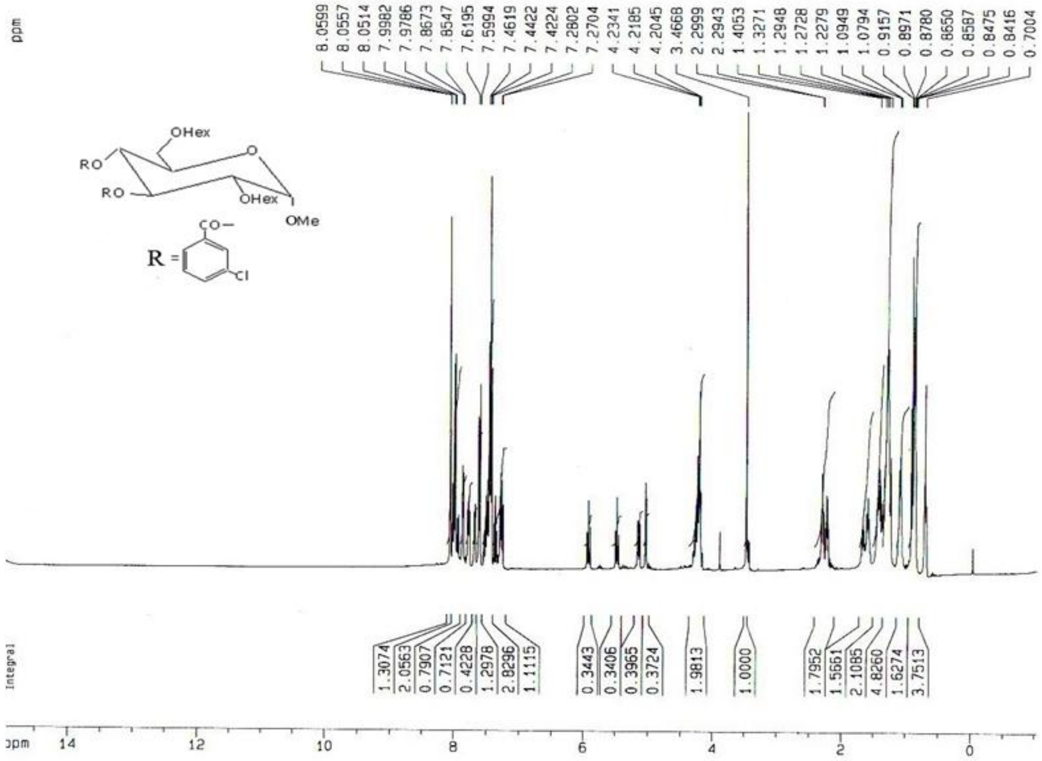

Fig. S11(A). ^{1}H NMR spectrum of compound **8**.

Article published in: Sci Pharm. 2014; 82: 1–20. doi:10.3797/scipharm.1308-03

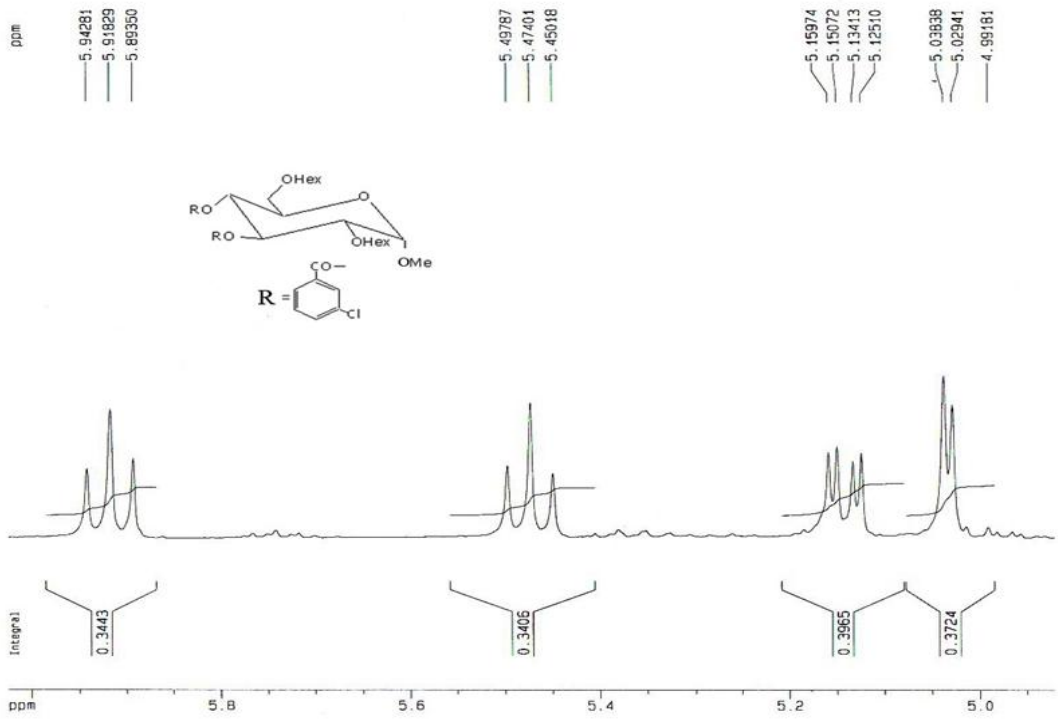

Fig. S11(B). ^{1}H NMR expansion of compound **8**.

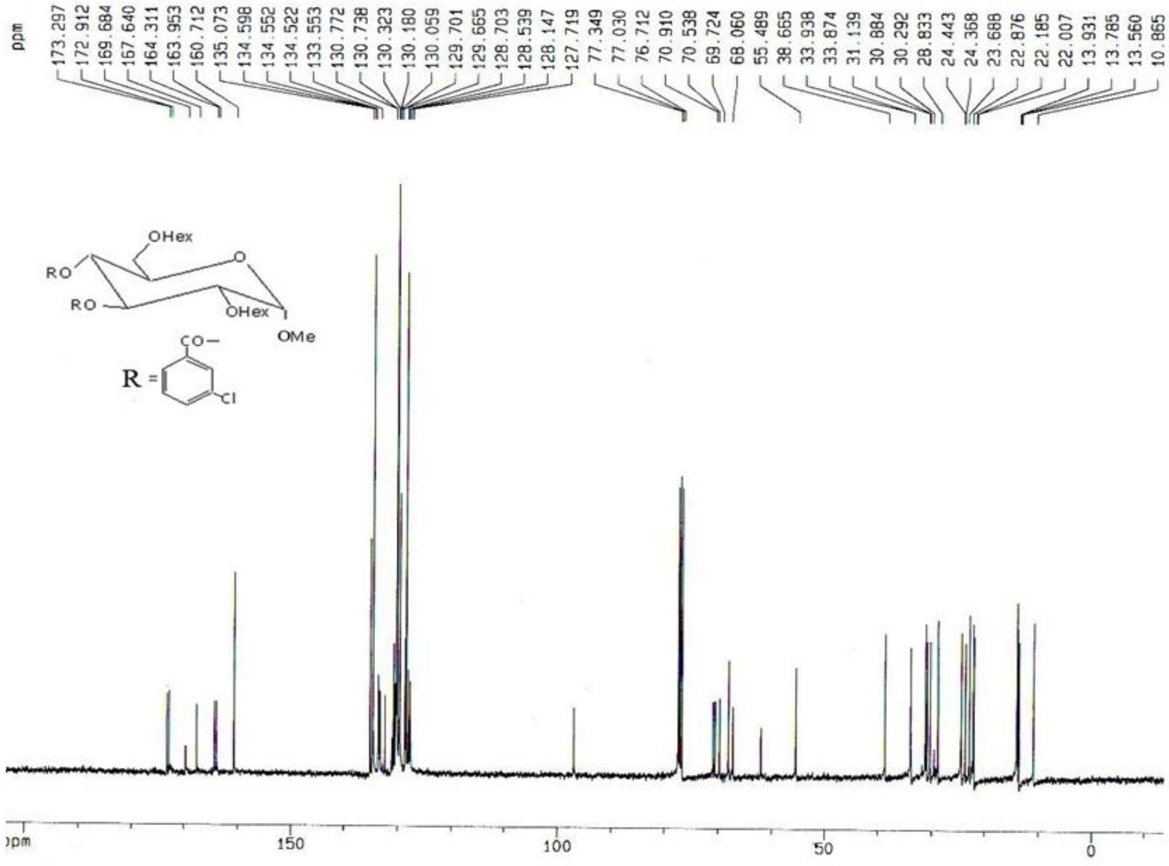

Fig. S12. ^{13}C NMR spectrum of compound **8**.

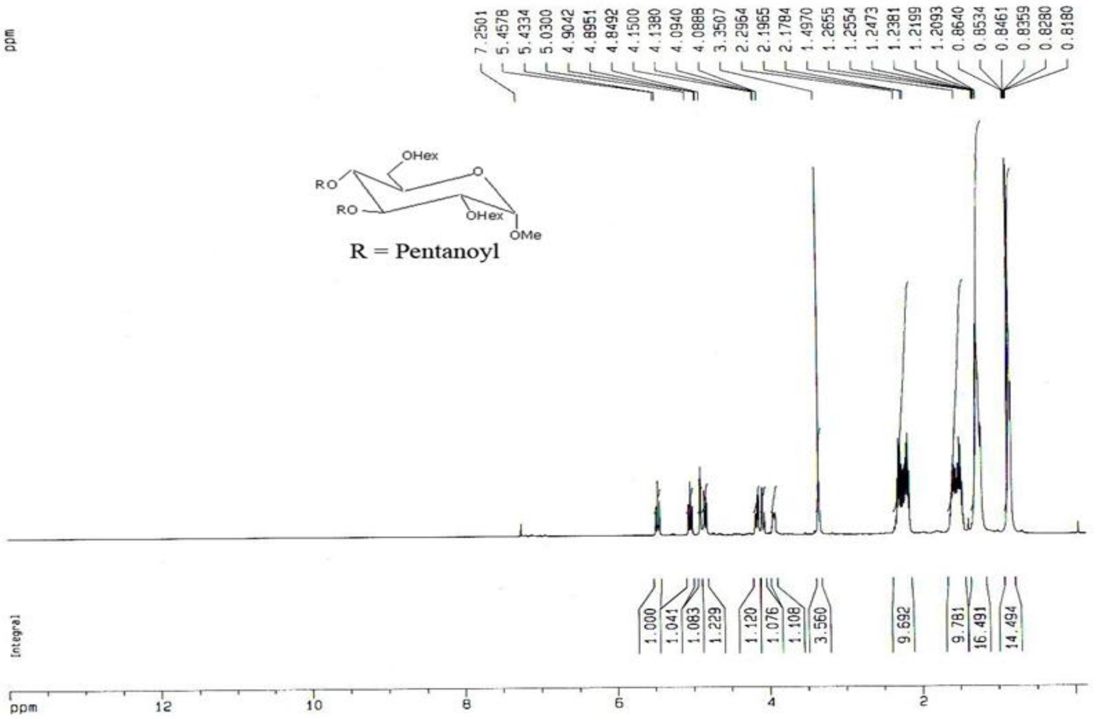

Fig. S13(A). ^{1}H NMR spectrum of compound **9**.

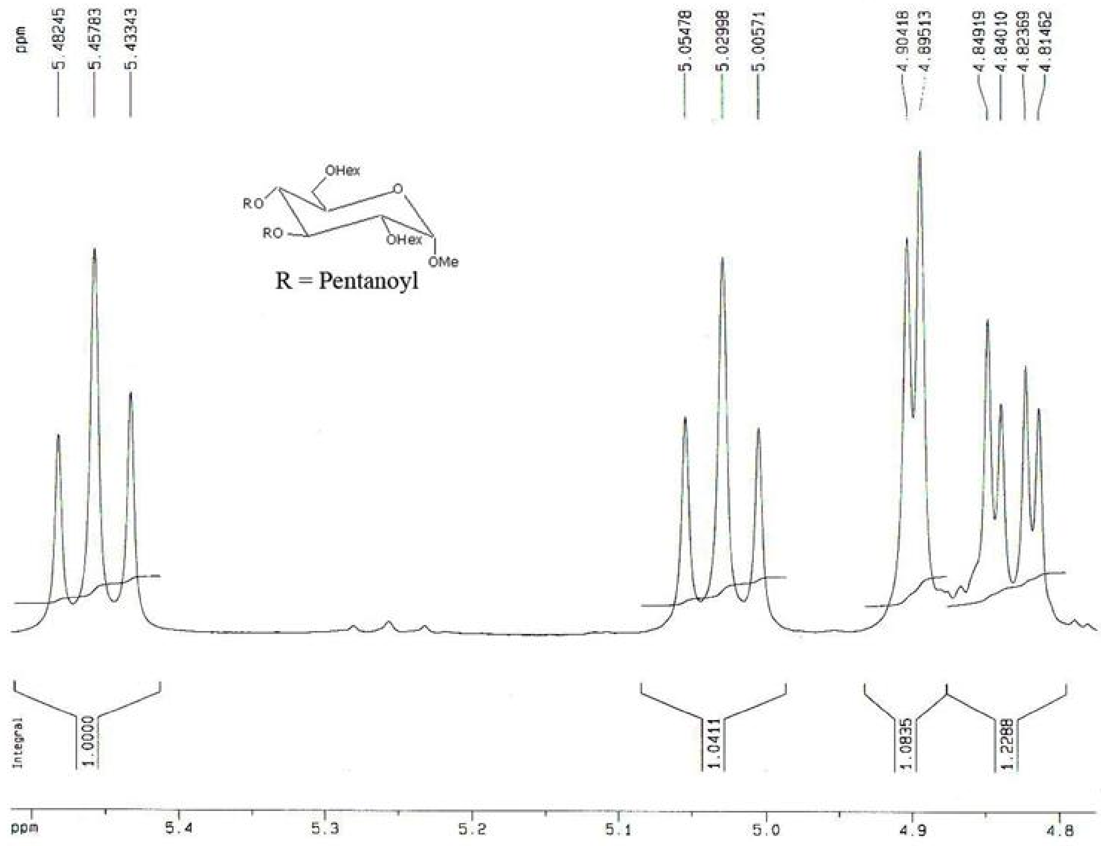

Fig. S13(B). ^{1}H NMR expansion of compound **9**.

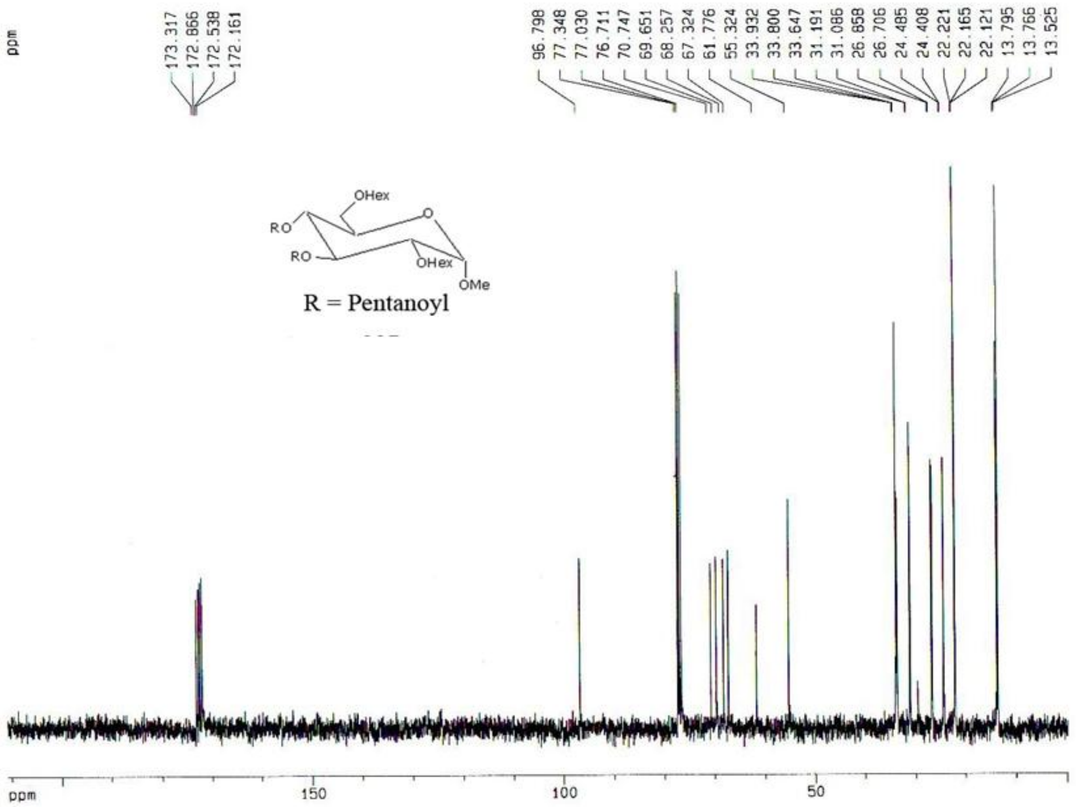

Fig. S14. ^{13}C NMR spectrum of compound **9**.

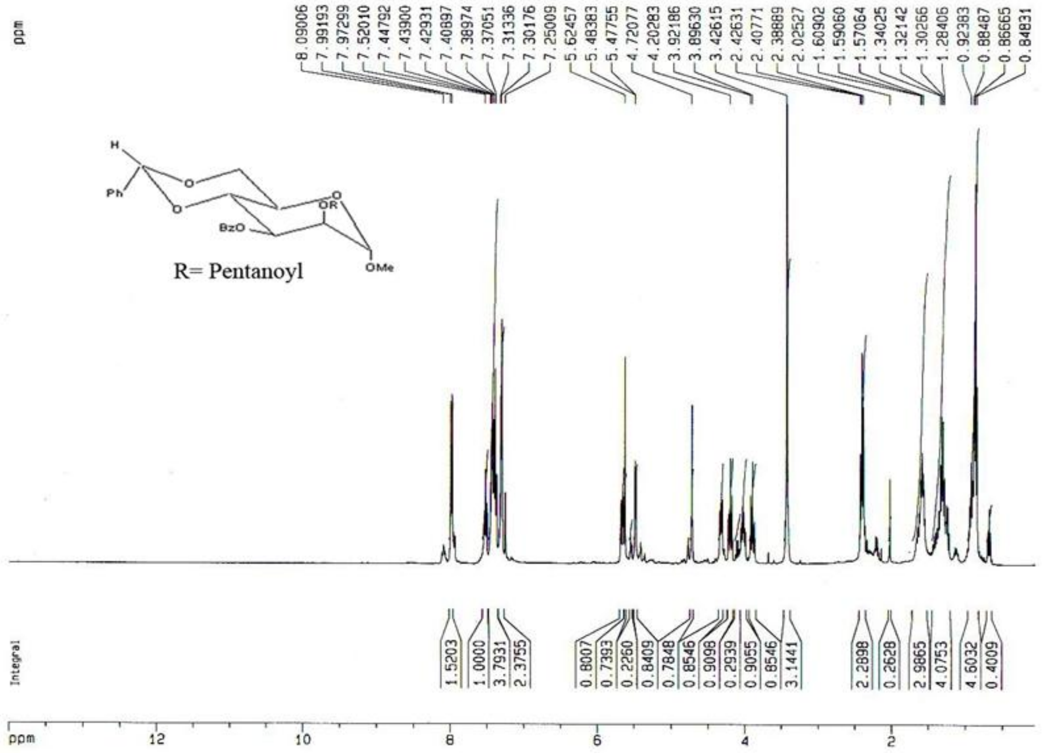

Fig. S15(A). ^{1}H NMR spectrum of compound **10**.

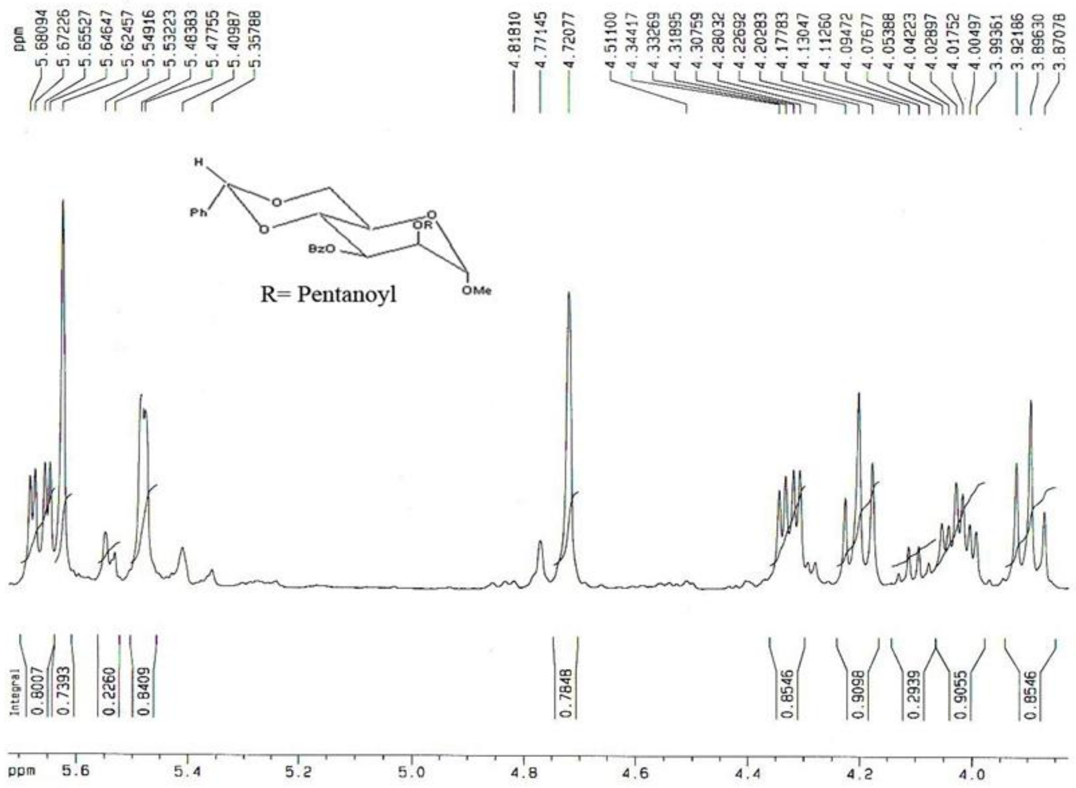

Fig. S15(B). ^{1}H NMR expansion of compound **10**.

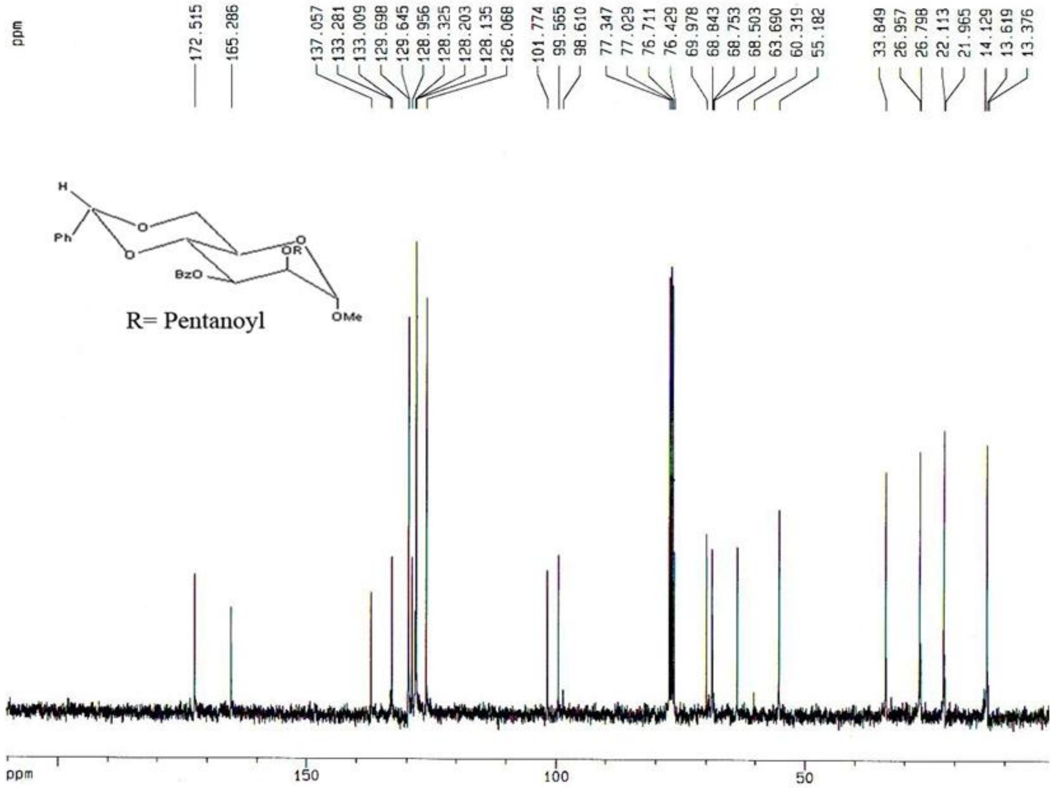

Fig. S16. ^{13}C NMR spectrum of compound **10**.

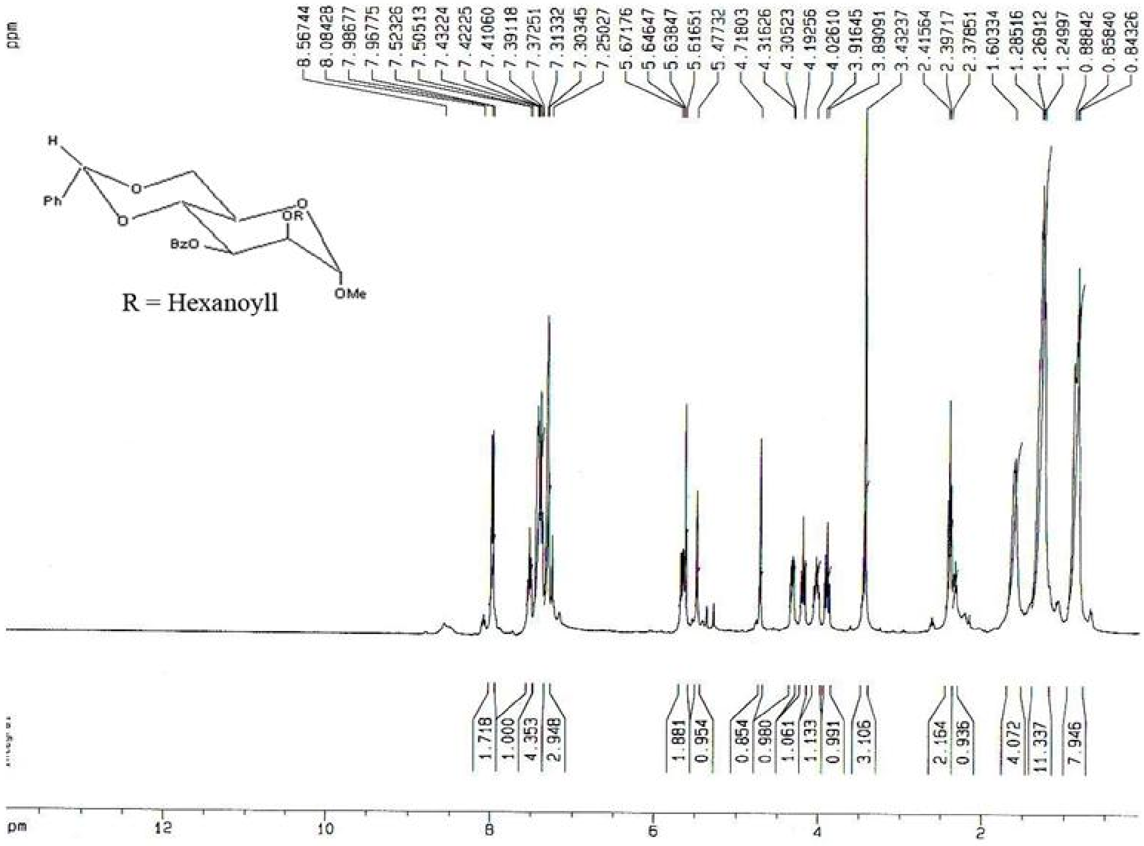

Fig. S17. ^{1}H NMR spectrum of compound **11**.

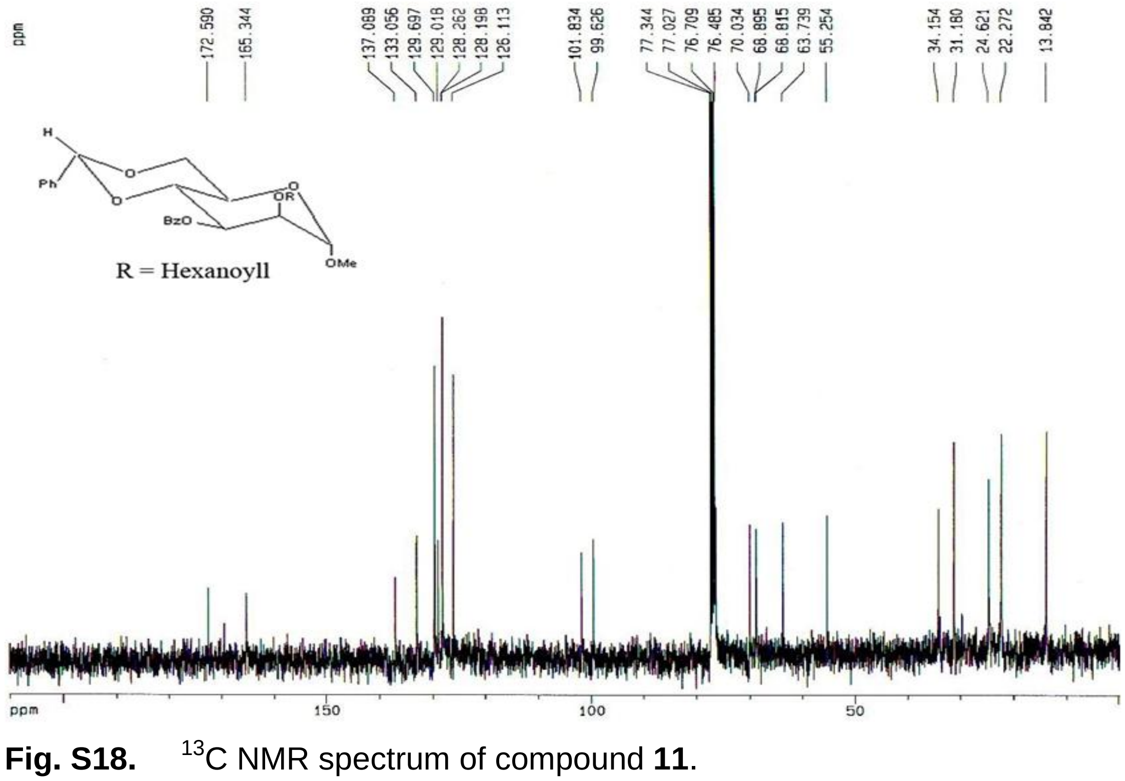

Fig. S18. ^{13}C NMR spectrum of compound **11**.

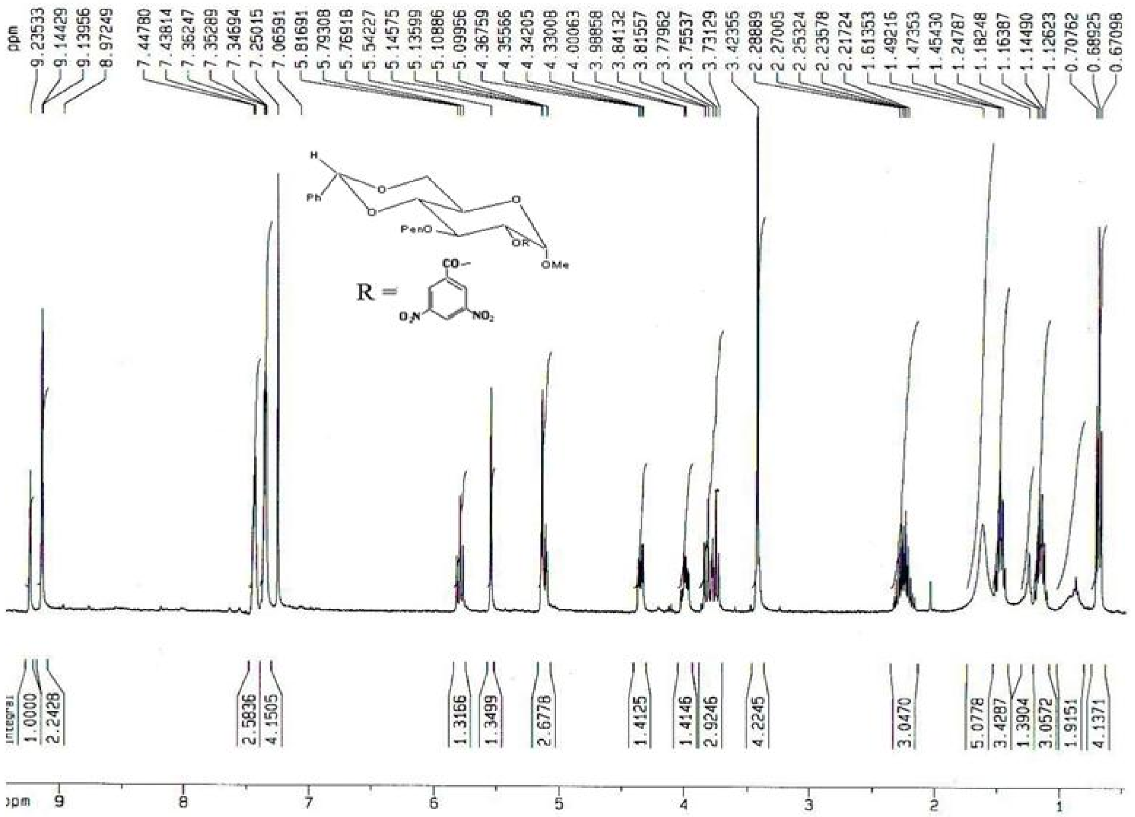

Fig. S19(A). ^{1}H NMR spectrum of compound **12**.

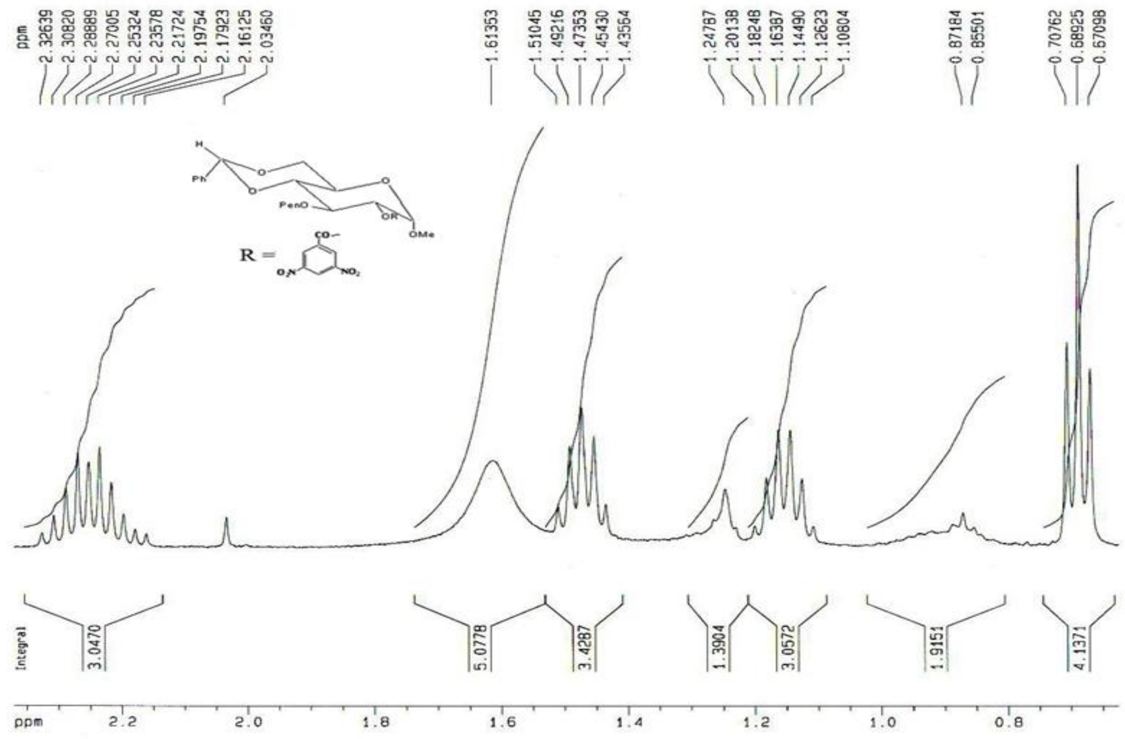

Fig. S19(B). ^{1}H NMR expansion of compound **12**.

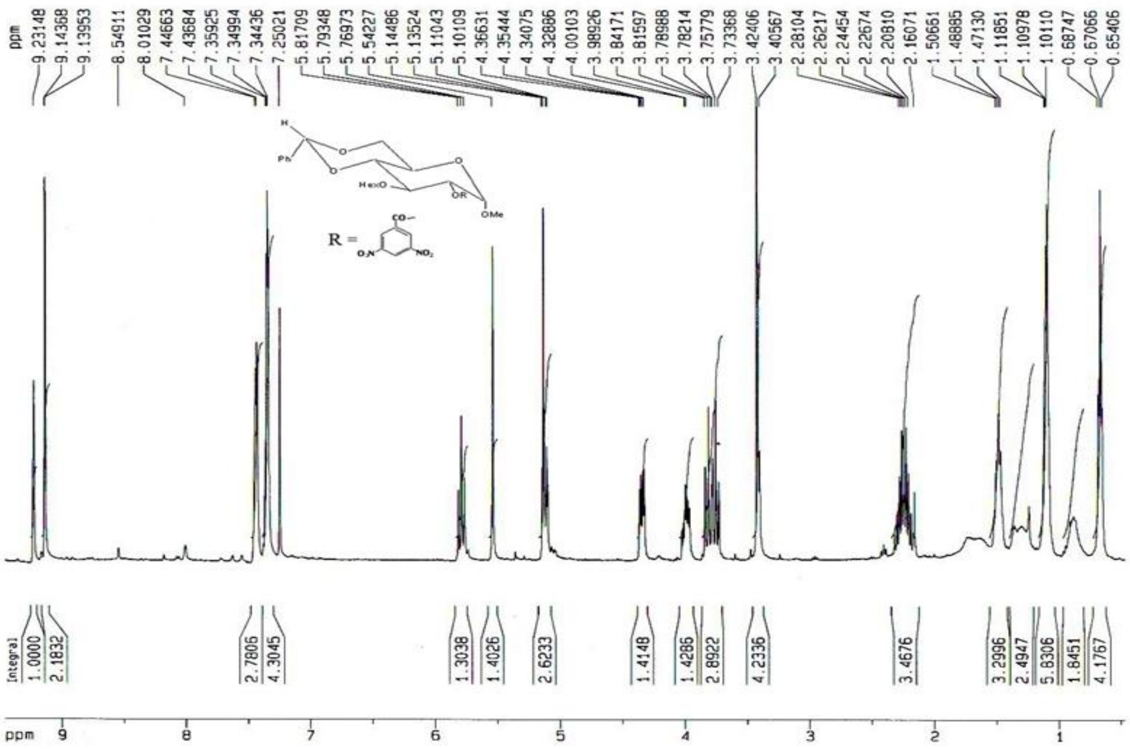

Fig. S20. ^{1}H NMR spectrum of compound **13**.

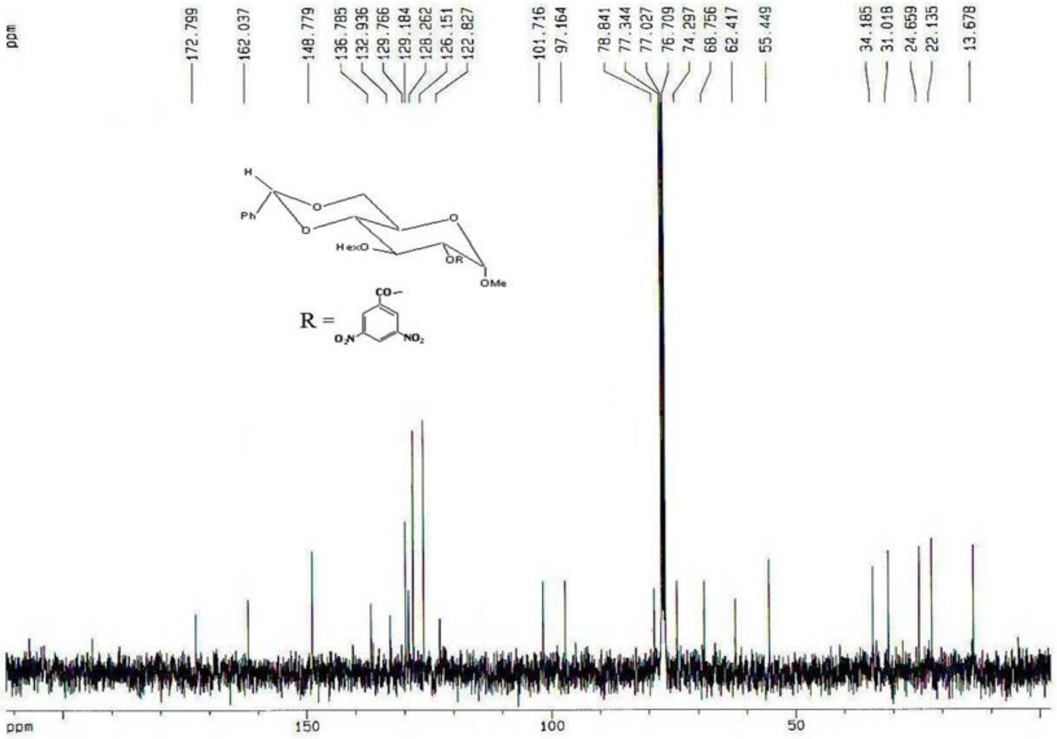

Fig. S21. ^{13}C NMR spectrum of compound **13**.
